# Supplementary material for: Water decontamination via nonradical process by nanoconfined Fenton-like catalysts
Source: Nat Commun. 2023 May 19;14:2881. doi: 10.1038/s41467-023-38677-1 (PMC10199022; doi:10.1038/s41467-023-38677-1)
Supplement: Supplementary file 1 — Supplementary Information [file 41467_2023_38677_MOESM1_ESM.pdf]

Supplementary Information

Water decontamination via nonradical process by nanoconfined Fenton-like catalysts

Tongcai Liu[1], Shaoze Xiao[1], Nan Li[1], Jiabin Chen[1, *], Xuefei Zhou[1], Yajie Qian[2], Ching-Hua Huang[3] and Yalei Zhang[1, *]

[1]*State Key Laboratory of Pollution Control and Resources Reuse, College of Environmental Science and Engineering, Tongji University, Shanghai 200092, PR China*

[2]*College of Environmental Science and Engineering, Donghua University, Shanghai, 201620, PR China*

[3]*School of Civil and Environmental Engineering, Georgia Institute of Technology, Atlanta, Georgia 30332, United States*

* Corresponding Authors.

E-mail: chenjiabincn@163.com (J. Chen),

zhangyalei@tongji.edu.cn (Y. Zhang).

Supplementary Note 1: Materials and chemicals.

Peracetic acid (~20% PAA, ~5% H_2O_2, and ~20% acetic acid (AA) by weight) was purchased from Sigma-Aldrich (St. Louis, MO, USA). Multiwalled carbon nanotubes (CNTs) (<d> = 10−20 nm and <l> = 10−30 μm) were purchased from XFNANO Nano Co., Ltd. (Nangjing, China). Hydrogen peroxide (30% w/w), peroxymonosulfate (PMS, $KHSO_5{\cdot}0.5KHSO_4{\cdot}0.5K_2SO_4$), peroxydisulfate (PDS), cobalt nitrate hexahydrate ($Co(NO_3)_2{\cdot}6H_2O$), bisphenol A (BPA), sulfamethoxazole (SMX), *p*-chlorophenol (4-CP), rhodamine B (RhB), imidacloprid (IMD), benzoic acid (BA), nitrobenzene (NB), atrazine (ATZ), carbamazepine (CBZ), furfuryl alcohol (FFA, ≥97.5%), methyl phenyl sulfoxide (PMSO), methyl phenyl sulfone ($PMSO_2$), sodium thiosulfate ($Na_2S_2O_3$), 2,4-hexadiene (2,4-HD), *Tert-* butyl alcohol (TBA), sodium azide (NaN_3), sodium chloride (NaCl), sodium bicarbonate ($NaHCO_3$), sodium sulphate (Na_2SO_4), sodium nitrate ($NaNO_3$) copper sulfate pentahydrate ($CuSO_4{\cdot}5H_2O$, AR, ≥ 99%), ferrous sulfate heptahydrate ($FeSO_4{\cdot}7H_2O$, AR, ≥ 99%), calcium chloride ($CaCl_2$, ACS, ≥ 96.0%), sodium hydroxide (NaOH), sulfuric acid (H_2SO_4), nitric acid (HNO_3), potassium iodide (KI), furfuryl alcohol (FFA), 9, 10-diphenylanthracene (DPA), dimethyl sulfoxide (DMSO), superoxide dismutase (SOD), N,N-diethyl-p-phenylenediamine (DPD), nitro blue tetrazolium (NBT), 5,5-Dimethyl-1-pyrroline N–Oxide (DMPO), 2,2,6,6-tetramethylpiperidine (TEMP) were purchased from Sigma-Aldrich (St. Louis, MO, USA) or Aladdin Reagent (Co., Shanghai, China) at analytical grade. 5-diisopropoxyphosphoryl-5-methyl-1-pyrroline N-oxide (DIPPMPO) was purchased from Enzo Biochem (Inc., New York, USA). Deuterium oxide (D_2O) was purchased

from J&K chemicals (Beijing, China). Methanol (MeOH) and acetonitrile (ACN) of high-performance liquid chromatography (HPLC) grade were purchased from Honeywell Burdick & Jackson (Ulsan, Korea). Humic acid (HA) was obtained from Shanghai Luzhong Chemical Reagent Co., Ltd (Shanghai, China) and used to represent natural organic matter (NOM). Unless otherwise specified, all chemicals were used as received without further purification. DPA stock solution was prepared using ACN as the solvent. All the stock solutions were prepared with ultrapure water (18.2 MΩ·cm) purified by a Milli-Q system, stored at 5°C before use.

Supplementary Note2: Preparation of catalysts.

Modification of CNTs: For the preparation of broken CNTs, the pristine CNTs were added to 200 mL of concentrated HNO_3 (68 wt%), the suspension was then sonicated for 1 h at room temperature to better disperse the particles. Afterwards, the mixture was refluxed at 403.2 ± 5 K for 14 h in an oil bath. Suction filtration was performed after cooling to room temperature, and the samples were washed with deionized water to neutral pH. Then, the samples were placed in 50 mL centrifuge tubes and ultrasonically dispersed for 30 min at room temperature. The samples were placed in a centrifuge (TDL-16M, China) and centrifuged at 7000 rpm for 10 min for better separation, and then was dried at 333.2 K in a vacuum desiccator (DZF-6020, Shsz, China) for 12 h. Finally, the centrifuge tubes were placed in a low temperature freezer (DW-25L-300, Aucma, China) for 30 min and then placed in a freeze dryer (SCIENTZ-10N, China) for lyophilization (228.2 K) with 48 h. The treatment of the

CNTs described above removed the caps of the nanotubes as well as the amorphous carbon and metal catalyst residues. For comparison, the purified CNTs were also prepared. Briefly, the pristine CNTs were first suspended in 200 mL of diluted HNO_3 solution (38 wt%) to remove residual metal impurities, and then refluxed at 382.2 ± 5 K in an oil bath under magnetic stirring for 14 h. After the mixture was cooled to room temperature, it was filtered through a PTFE membrane with a pore size of 0.2 μm, and washed with deionized water until the pH of the filtrate solution at neutral pH. The product was dried in a vacuum oven at 333.2 K for 12 h.

Fabrication of catalysts: The catalysts of Co_3O_4-*in*-CNT and Co_3O_4-*out*-CNT were prepared through chemical vapor deposition. $Co(NO_3)_2 \cdot 6H_2O$ (0.3 g, 51.54 mM) was dissolved in 20 mL 99 wt% EtOH to serve as an cobalt precursor. Afterwards, each of 0.2 g supporter (i.e., purified CNTs, broken CNTs) was dispersed in deionized water by sonication for 1 h, respectively. The supporter (CNTs) and cobalt nitrate hexahydrate were first mixed in acetone solution for 1 h and then sonicated for 4 h, which was introduced into the CNTs channels utilizing the capillary forces of CNTs aided by ultrasonic and stirring treatment. Then the solvent was evaporated in a vacuum oven at 353.2 K for 12 h. The solid mixture samples were gradually heated to 623.2 K (heating rate: 274.2 K min^{-1}) in air and maintained for 2 h in tubular furnace (OTF-1200X, China). Afterwards, the solid sample at room temperature was washed with EtOH and deionized water alternately for 5–6 times and then placed in a vacuum oven at 353.2 K for 8 h. $Co(NO_3)_2 \cdot 6H_2O$ decomposes inside broken CNTs into Co_3O_4 and the obtained sample is denoted as Co_3O_4-*in*-CNT. For comparison, the same

procedure of Co_3O_4 was deposited on the outer surface of nanotubes by impregnating purified CNTs with aqueous $Co(NO_3)_2 \cdot 6H_2O$ solution. In this process, the pristine CNTs were first suspended in diluted HNO_3 solution (38 wt%), and then refluxed at 382.2 ± 5 K in an oil bath g for 14 h.

Supplementary Note 3: Analytical methods of organic compounds.

Target contaminant (BPA) was determined on a HPLC system (1200, Agilent Technology, USA) equipped with a C18 column (4.6 × 250 mm, 5 μm). The mobile phase was methanol/water (70:30, v/v%) with a flow rate of 1.0 mL min^{-1}, and the detection wavelength was selected at 280 nm. The concentrations of PMSO and $PMSO_2$ were analyzed using an Agilent series 1200 HPLC with UV detection equipped with an C18 column (4.6 × 250 mm, 5 μm). The mobile phase consisted of 80% water (containing 0.1% acetic acid) and 20% acetonitrile at a flow rate of 1 mL min^{-1}. The detection wavelengths for PMSO and $PMSO_2$ were set at 230 and 215 nm, respectively. The residual RhB concentration was analyzed at the maximum absorption wavelength by the UV-vis spectrophotometer. Detection details for other organic contaminants are provided in Supplementary Table 1. The transformation byproducts of BPA were identified by UPLC system (1290UPLC, Agilent, USA) connected with a triple quadrupole mass spectrometer (QTOF6550, Agilent, USA). BPA was separated on a Zorbax SB-C18 column (2.1 × 150 mm, 5 μm). The mobile phase A was water with formic acid (0.1%, v/v) and the mobile phase B was 100% acetonitrile. The mobile phase B was increased from 20% to 80% in 10 min and kept

for 4 min. Afterwards, the mobile phase B decreased from 80% to 20% in 14.1 min and kept for 6 min. MS and MS/MS patterns of BPA and TPs were analyzed with an m/z range of 50 to 500 in negative electrospray ionization (ESI) modes, sheath gas temperature 350 ℃ and sheath gas flow 12 L min^{-1}.

DPA stock solution was prepared using 50 mM ACN. The reaction aliquot was withdrawn at the end of the reaction (30 min) and filtered through a PTFE membrane (0.22 μm) to remove the solid catalysts for analysis. HPLC/MS/MS with an ESI source in the positive ionization mode was used for analysis. An Agilent Eclipse plus C18 column (4.6 × 100 mm, 3.5 μm particle size) was used for HPLC separation. The flow rate was set at 0.2 mL·min^{-1}. To eliminate the possible contamination of mass spectrometer by the oxidants and other impurities in the samples, the HPLC effluent in the first 2 min was diverted to the waste through a switching valve between the HPLC column outlet and the mass detector inlet. The gradient mobile phase consisted of ACN (A) and Milli-Q water (B), which increased linearly from 65/35 to 95/5 (v/v, A/B) in first 25 min and held for 10 min, and then returned to 65/35 in 0.5 min, and kept for 5 min for re-equilibration. The MS parameters were optimized and set as follows: scan type, full mass; resolution, 35000; sheath gas flow rate, 20 arbitrary units; aux gas flow rate, 10 arbitrary units; spray voltage, 3000 V; capillary temperature, 593.2 K.

Supplementary Note 4: Determination of PAA concentration.

The concentration of PAA was determined by the

N,N-diethyl-p-phenylenediamine (DPD) colorimetric method [1]. We know that the PAA stock solution contains PAA and H_2O_2. Hence, the sum of the concentration of PAA and H_2O_2 was first determined by iodimetry titration. Briefly, potassium iodide was added as an iodine source, and ammonium molybdate was used as a catalyst to generate free iodine. The total oxidants concentration in the system was then determined by titration of free iodine with sodium thiosulfate. Afterwards, the H_2O_2 concentration in the solution was titrated with potassium permanganate under acidic pH. Hence, the concentration of PAA in the stock solution was obtained by subtracting the H_2O_2 concentration from the total oxidants' concentration.

PAA working solution at 10 gL^{-1} was prepared weekly by diluting the standardized PAA stock solution and then stored at 5°C. The N, N-diethyl-p-phenylenediamine (DPD) colorimetric method was used to determine the residual PAA concentration. The sample were periodically collected and immediately treated with excess amount of potassium iodide, then PAA reacted with potassium iodide to produce iodine. The iodine produced above was further reacted with DPD to form a pink-colored species, the sample was analyzed at the maximum absorption wavelength of 515 nm by the UV-vis spectrophotometer [2].

Supplementary Note 5: Performance of Fenton-like reactions under spatial nanoconfinement.

To assess the effect of spatial nanoconfinement on tuning various oxidants (i.e., PAA, PMS, PDS and H_2O_2) activation, the Fenton-like catalytic performance for BPA

removal were further investigated. As shown in Supplementary Figure 9, noticeable BPA degradation was observed in coupling of Co_3O_4-CNTs with various peroxides, indicating the effectiveness of Co_3O_4-CNTs on the activation of various peroxides. Generally, the observed rate constant (k_{obs}) of BPA in the Co_3O_4-*in*-CNT/peroxides were higher than those in the Co_3O_4-*out*-CNT/peroxides, also indicating that Co_3O_4-*in*-CNT was more efficient to activate peroxides. Specifically, PAA and PMS are more effectively activated by Co_3O_4-*in*-CNT than PDS and H_2O_2**,** which was due to the unsymmetrical molecular structure (Supplementary Table 4).[3,4] In addition, the lower bond dissociation energy of PAA (PAA: 159 kJ·mol^{-1}, H_2O_2: 213 kJ·mol^{-1}, PMS: 377 kJ·mol^{-1}) was also conducive to the activation of peroxide bond.[5,6] . Therefore, it can be concluded that the Co_3O_4-*in*-CNT/oxidants is robust Fenton-like reactions with potentially generic applicability. Importantly, PAA based Fenton-like system has received increasing attention for the degradation of recalcitrant pollutants in water because of the strong oxidizing capability of organic radicals (R-O$^•$, e.g., acetoxy(per) radicals ($CH_3C(O)O^•$ and $CH_3C(O)OO^•$) at wider pH ranges and the low potential to form harmful disinfection byproduct [1,7,8,9,10].

Supplementary Note 6: Radicals production during PAA activation.

The cleavage of organic peroxide bond in PAA produces reactive radicals with strong oxidation capacity, e.g., R-O$^•$ or ROS [2,6,9,11,12,13,14,15]. HO$^•$ and R-O$^•$, including $CH_3C(O)O^•$, $CH_3C(O)OO^•$, $CH_3OO^•$ and $CH_3^•$ (eqs S1-8), can be generated through PAA activation by energy transfer (e.g., UV irradiation) or electron transfer

(e.g., heterogeneous catalyst). Therefore, the generation process of organic radicals was identified in the reaction system.

$$CH_3C(O)OOH \Leftrightarrow CH_3C(O)O^\bullet + HO^\bullet \quad (S1)$$

$$CH_3C(O)O^\bullet \rightarrow CH_3^\bullet + CO_2 \quad (S2)$$

$$CH_3C(O)O^\bullet + CH_3C(O)O^\bullet \rightarrow (CH_3C(O)O)_2 \quad (S3)$$

$$CH_3^\bullet + O_2 \rightarrow CH_3OO^\bullet \quad (S4)$$

$$CH_3C(O)OOH + HO^\bullet \rightarrow CH_3C(O)OO^\bullet + H_2O \quad (S5)$$

$$CH_3C(O)OOH+CH_3C(O)O^\bullet \rightarrow CH_3C(O)OO^\bullet + CH_3C(O)OH \quad (S6)$$

$$CH_3C(O)OO^\bullet \rightarrow CH_2CO + O_2^{\bullet-} + H^+ \quad (S7)$$

$$CH_3C(O)O^\bullet + H_2O_2 \rightarrow O_2^{\bullet-} + CH_3C(O)OH + H^+ \quad (S8)$$

Supplementary Note 7: EPR analysis for R-O$^\bullet$.

EPR was employed to detect reactive species at ambient temperature in our systems. Specifically, 2,2,6,6-tetramethyl- 4-piperidinol (TEMP) (100 mM) were used as $^1O_2$ spin-trapping agents with the formation of 2,2,6,6-tetramethyl-4-piperidinol-N-oxyl (TEMPO) adduct. In addition, 5,5-dimethyl-1-pyrroline-*N*-oxide (DMPO) (100 mM) was used as spin-trapping agents to identify HO$^\bullet$ and $O_2^{\bullet-}$. The EPR spectra were monitored using an EPR spectrometer (Bruker, Germany), with the instrument settings as follows: modulation frequency of 100 kHz, modulation amplitude of 1 G, microwave power of 0.2 mW, sweep width of 200 G, and sweep time of 167 s.

In addition, 5-diisopropoxyphosphoryl-5-methyl-1-pyrroline N-oxide

(DIPPMPO) was used as a trapping agent for R-O• analysis [6, 7]. Then, the EPR spectra were simulated to extract the values of hyperfine splitting constants (α_N, α_H and α_P) for radical identification with MATLAB (Figure 2c). The simulated results indicated the existence of HO•, $CH_3C(O)O^•$, $CH_3^•$ and $CH_3OO^•$ adducts in the Co_3O_4-*out*-CNT/PAA system (Supplementary Table 5). Specifically, the trapping adducts for HO• (8-line signal, marked by green dots), $CH_3^•$ (12-line signal, marked by yellow triangles), and $CH_3OO^•$ (12-line signal, marked by red stars) were clearly observed. The $CH_3C(O)OO^•$ adduct was not simulated in the EPR spectra, which might be attributed to the high instability of this radical and the lack of related fitting information [6, 7]. Although the redox potential of $CH_3C(O)O^•$ (E^0 = 2.24 V) is higher than that of $CH_3C(O)OO^•$ (E^0 = 1.60 V), the self-dissociation of $CH_3C(O)O^•$ (eq S2, 2.3×10^5 s^{-1}) and the quenching reaction by itself (eq S3, 1.0×10^9 M^{-1} s^{-1}) or PAA (eq S6, 10^7 M^{-1} s^{-1}), induced its rapid decomposition after generation [1]. In addition, the oxidation of BPA by $CH_3C(O)O^•$ is calculated to be negligible ($<$ 4%) (Supplementary Note 8). The self-decomposition of $CH_3C(O)O^•$ via β-cleavage reaction produce $CH_3^•$, which could further react with O_2 to generate $CH_3OO^•$ ($k = 4.1 \times 10^9$ M^{-1} s^{-1}). [1, 11] Nevertheless, both $CH_3OO^•$ and $CH_3^•$ exhibit weak oxidation capacity, and their contribution to BPA degradation could be neglected (Supplementary Note 8). Hence, $CH_3C(O)OO^•$ was assumed as the primary reactive species responsible for contaminant degradation in the Co_3O_4-*out*-CNT/PAA system.

Supplementary Note 8: Contributions of $CH_3C(O)O^•$, $CH_3^•$ and $CH_3OO^•$ for BPA

degradation.

The direct oxidation of $CH_3C(O)O^•$ on BPA degradation: According to quenching experiments and EPR characterization, $CH_3C(O)O^•$ was indeed generated in the Co_3O_4-*out*-CNT/PAA system. Nevertheless, $CH_3C(O)O^•$ could also be consumed in the following reactions. For instance, $CH_3C(O)O^•$ could be consumed by the self-decay reaction (eq S2), which is a typical β-cleavage reaction. In addition, $CH_3C(O)O^•$ could also react with PAA, BPA and other compounds in the system. Hence, the $CH_3C(O)O^•$ concentration as a function of reaction time is shown in eq S9.

$$\frac{d[CH_3C(O)O^•]}{dt} = (k_{\beta-cleavage} + k_{PAA,CH3C(O)O•}[PAA] + k_{BPA,CH3C(O)O•}[BPA] + \sum_i k_i [s_i])[CH_3C(O)O^•] \quad (S9)$$

Thus, the fraction of $CH_3C(O)O^•$ reacting with BPA can be described as eq S10:

$$f_{BPA,CH3C(O)O•}(\%) = \frac{k_{BPA,CH3C(O)O•}[BPA]}{k_{\beta-cleavage} + k_{PAA,CH3C(O)O•}[PAA] + k_{BPA,CH3C(O)O•}[BPA] + \sum_i k_i [s_i]} \le \frac{k_{BPA,CH3C(O)O•}[BPA]}{k_{\beta-cleavage} + k_{PAA,CH3C(O)O•}[PAA] + k_{BPA,CH3C(O)O•}[BPA]} = f_{max} \quad (S10)$$

where $k_{\beta\text{-cleavage}}$ represents the reaction rate constant for the self-decay of $CH_3C(O)O^•$. However, the values of $k_{\beta\text{-cleavage}}$ reported in the previous literature to date vary considerably from different studies [16], including 1.0×10^5 s^{-1} at room temperature, 1.0×10^6 s^{-1}, < 1.3×10^9 s^{-1} (20 °C), 6×10^9 s^{-1} (60 °C) and 10^9–10^{10} s^{-1} (65 °C) [16]. The values of $k_{PAA,CH3C(O)O•}$, $k_{BPA,CH3C(O)O•}$ and k_i are the second order constants (M^{-1} s^{-1}) of $CH_3C(O)O^•$ with PAA, BPA and other compounds, respectively. According to previous reports, the $k_{PAA,CH3C(O)O•}$ value was 10^7 M^{-1} s^{-1}, and the reaction rate constant of $CH_3C(O)O^•$ with BPA ($k_{BPA,CH3C(O)O•}$) reached 10^7-10^8 M^{-1} s^{-1}. In our experimental conditions, the concentrations of BPA and PAA are 100 and 200 μM,

respectively. Therefore, if the lowest reported value (10^5 s^{-1}) is selected, the fraction of $CH_3C(O)O^•$ reacting with BPA could be no more than 0.97% - 3.9%. However, if a high $k_{β\text{-cleavage}}$ value (10^9 s^{-1}) was selected, the fraction of $CH_3C(O)O^•$ reacting with BPA could be ignored because almost all $CH_3C(O)O^•$ was consumed by its self-decay reaction. Overall, the oxidative contribution of $CH_3C(O)O^•$ to BPA is small through above analysis in the Co_3O_4-*out*-CNT/PAA system. Hence, the oxidation of BPA by $CH_3C(O)O^•$ could be neglected in the Co_3O_4-*out*-CNT/PAA system.

The contributions of $CH_3^•$ and $CH_3OO^•$: The self-dissociation of $CH_3C(O)O^•$ could produce $CH_3^•$, which further rapidly react with O_2 to produce $CH_3OO^•$. Hence, the role of $CH_3^•$ and $CH_3OO^•$ on BPA degradation in Co_3O_4-*out*-CNT/PAA process was evaluated. The experiments were performed in oxygen-deprived and oxygen-saturated conditions via purging N_2 and O_2 in the Co_3O_4-*out*-CNT/PAA+TBA system, respectively. Meanwhile, the contribution of $HO^•$ could be exclude with the addition of TBA, thus the primarily radicals were $CH_3C(O)O^•$, $CH_3C(O)OO^•$, $CH_3^•$ and $CH_3OO^•$ in the system. As shown in Supplementary Figure 11, the removal of BPA was slightly affected under anoxic and oxygen-enriched conditions, implying negligible contributions of $CH_3^•$ and $CH_3OO^•$ in the Co_3O_4-*out*-CNT/PAA system. Overall, the above evidences verified $CH_3C(O)OO^•$ as the primary reactive species responsible for BPA degradation in the Co_3O_4-*out*-CNT/PAA system.

Supplementary Note 9: Kinetic solvent isotope effect (KSIE) experiments.

The kinetic solvent isotope effect (KSIE) was performed to verify that $^1O_2$ is

produced in the process by changing the solvent from H_2O to deuterium oxide (D_2O) [4, 17]. The oxidative coupling and polymerization routes consist of H rearrangement and H leaving steps, which involve the participation of solvent H_2O. Therefore, the solvent H_2O was replaced by D_2O in the experiments to explore the KSIE on the reaction rate [18].

Supplementary Note 10: Effect of $O_2^{\bullet-}$ on the degradation of BPA.

The role of $O_2^{\bullet-}$ on pollutants degradation were always ignored due to the low reactivity (E_h^0 = 0.33 V) in PAA activation systems (e.g., activation by Co(II) or heat)[11, 12]. The production of $O_2^{\bullet-}$ has been confirmed according to EPR experiments, however whether it directly degrades BPA is largely unknown in our system. Hence, the contribution of $O_2^{\bullet-}$ was further investigated to accurately reveal the reaction mechanism in the Co_3O_4-*in*-CNT/PAA system. Firstly, the photochemical generation of $O_2^{\bullet-}$ in ketone/alcohol solutions was developed [19, 20, 21, 22]. Briefly, the $O_2^{\bullet-}$-containing solutions was prepared by the photolysis of oxygen-saturated solutions with 41.0 mM acetone, 12.0 M 2-propanol, 15.0 µM diethylenetriaminepentaacetic acid (DTPA) and 1.0 mM borate buffer [1]. The reaction process is shown in the following eq S11-S14.

$$(CH_3)_2CO \xrightarrow{hv} (CH_3)_2C\text{-}O^3 \quad (S11)$$

$$(CH_3)_2C\text{-}O^3 + (CH_3)_2CHOH \rightarrow 2{\bullet}C(CH_3)_2OH \quad (S12)$$

$$\bullet C(CH_3)_2OH + O_2 \rightarrow \bullet OOC(CH_3)_2OH \quad (S13)$$

$$\bullet OOC(CH_3)_2OH + HO^- \rightarrow (CH_3)_2CO + H_2O + O_2^{\bullet-} \quad (S14)$$

Afterwards, the second-order rate constants of BPA with $O_2^{\bullet-}$ in the $O_2^{\bullet-}$-containing solutions was determine by competition kinetic method (Supplementary Figure 14). Phenol ($k_{O_2^{\bullet-}, \text{phenol}} = (2.7 \pm 1.2) \times 10^3$ M^{-1} s^{-1}) was used as the competitor compound [23]. The degradation of phenol in the system was attributed to direct photolysis and $O_2^{\bullet-}$ oxidation (eq S15).

$$-\frac{d[\text{phenol}]}{dt} = k_{\text{obs,phenol}}[\text{phenol}]$$
$$= k_{\text{d,phenol}}[\text{phenol}] + k_{\text{i,phenol}}[\text{phenol}]$$
$$= k_{\text{d,phenol}}[\text{phenol}] + k_{O_2^{\bullet-},\text{phenol}}[O_2^{\bullet-}][\text{phenol}] \quad (S15)$$

Similarly, the degradation of BPA in the $O_2^{\bullet-}$-containing solutions could be expressed by the eq S16:

$$-\frac{d[\text{BPA}]}{dt} = k_{\text{obs,BPA}}[\text{BPA}]$$
$$= k_{\text{d,BPA}}[\text{BPA}] + k_{\text{i,BPA}}[\text{BPA}]$$
$$= k_{\text{d,BPA}}[\text{BPA}] + k_{O_2^{\bullet-},\text{BPA}}[O_2^{\bullet-}][\text{BPA}] \quad (S16)$$

where $k_{\text{obs,phenol}}$ and $k_{\text{obs,BPA}}$ were the observed pseudo-first-order degradation rate constants (s^{-1}); $k_{\text{d,phenol}}$ and $k_{\text{d,BPA}}$ were the measured pseudo-first-order direct photolysis rate constants (s^{-1}); $k_{\text{i,phenol}}$ and $k_{\text{i,BPA}}$ were the indirect photolysis rate constants (s^{-1}); $k_{O_2^{\bullet-},\text{phenol}}$ and $k_{O_2^{\bullet-},\text{BPA}}$ were the second-order rate constant (M^{-1} s^{-1}) with $O_2^{\bullet-}$ of the phenol and BPA, respectively. The $k_{O_2^{\bullet-},\text{BPA}}$ was determined through eq S17 with the copresence of phenol and BPA in the $O_2^{\bullet-}$-containing solutions.

$$\left(\ln\left(\frac{[\text{BPA}]_t}{[\text{BPA}]_0}\right) - k_{\text{d,BPA}} * t\right) / \left(\ln\left(\frac{[\text{phenol}]_t}{[\text{phenol}]_0}\right) - k_{\text{d,phenol}} * t\right) = k_{O_2^{\bullet-}, \text{BPA}} / k_{O_2^{\bullet-}, \text{phenol}} \quad (S17)$$

where $[\text{BPA}]_t$ and $[\text{phenol}]_t$ are the initial concentrations of BPA and phenol, respectively; $k_{O_2^{\bullet-}, \text{BPA}}$ and $k_{O_2^{\bullet-}, \text{phenol}}$ are the second-order rate constants of BPA and

reference compound phenol for $O_2^{\bullet-}$. Accordingly, $k_{O_2^{\bullet-}, BPA}$ was determined to be 3.2×10^3 M^{-1} s^{-1}.

The contribution of $O_2^{\bullet-}$ to the degradation of BPA was further investigated in the Co_3O_4-*in*-CNT/PAA system. $O_2^{\bullet-}$ has been confirmed to indeed produce in the system based on EPR analysis and quenching experiments. Meanwhile, it also can be consumed through reaction with BPA ($k = 3.2 \times 10^3$ M^{-1} s^{-1}), Co(II) (10^8 -10^9 M^{-1} s^{-1}) and the decay of $O_2^{\bullet-}$ in self-termination reactions ($k = 9.7 \times 10^9$ M^{-1} s^{-1}) [23]. Thus, the fraction of $O_2^{\bullet-}$ reacting with BPA can be described as eq S18:

$$f_{O_2\bullet-,BPA} = \frac{k_{O_2\bullet-,BPA}[BPA]}{k_{O_2\bullet-,BPA}[BPA] + k_{self}[O_2\bullet-] + \sum_i k_{i,}[C]i} \leq \frac{k_{O_2\bullet-,BPA}[BPA]}{k_{O_2\bullet-,BPA}[BPA] + k_{self}[O_2\bullet-]} \quad (S18)$$

Therefore, the contribution of $O_2^{\bullet-}$ to BPA degradation could be ignored in the Co_3O_4-*in*-CNT/PAA system. However, EPR analysis and NBT probe experiments have indeed detected $O_2^{\bullet-}$ in the solution, thus $O_2^{\bullet-}$ was assumed to primarily participate in the ROS transformation process.

Supplementary Note 11: Evidence of the formation of $^1O_2$.

The intermediate products from the oxidation of DPA: The 9, 10-diphenylanthracene (DPA) is extensively used as $^1O_2$ specific capture probe [4, 24]. DPA rapidly reacts with $^1O_2$ at a reaction rate of $k = 1.3 \times 10^6$ M^{-1} s^{-1} to form stable DPA endoperoxide ($DPAO_2$) [25]. The HPLC/MS/MS at multiple reaction monitoring (MRM) mode was employed to analyze the intermediate product of DPA

(Supplementary Note 3). As shown in Figure 2e, the chromatographic peak of $DPAO_2$ was observed in the Co_3O_4-*in*-CNT/PAA solution. Furthermore, the concentration of $DPAO_2$ was significantly reduced with addition of BPA, indicating the production of $^1O_2$ as well as the degradation of BPA.

The intermediate products from the oxidation of FFA: Furfuryl alcohol (FFA, $C_5H_6O_2$) was previously reported to react with $^1O_2$ to produce three typical products (eq S19) [24]. Thus, relatively high concentration of FFA (1 or 5 mM) was selected for the determination of $^1O_2$ in the Co_3O_4-*in*-CNT/PAA system. Nevertheless, the intermediate products (*i.e.*, $C_5H_6O_4$, $C_4H_4O_3$, and $C_5H_6O_3$) from the oxidation of FFA by $^1O_2$ is commercially unavailable. Hence, the concentration of the products by HPLC peak area was calculated on the basis of the molar adsorption coefficient with FFA as a reference [24, 26]. In addition, the molar adsorption coefficient of FFA, $C_5H_6O_4$, $C_4H_4O_3$, and $C_5H_6O_3$ were 1320 ± 40, 2000, 7500 ± 200, and 8100 ± 60 M^{-1} cm^{-1}, respectively. Accordingly, the concentration of $C_5H_6O_4$, $C_4H_4O_3$ and $C_5H_6O_3$ was calculated on the basis of the Lambert-Beer law (eqs S20-22).

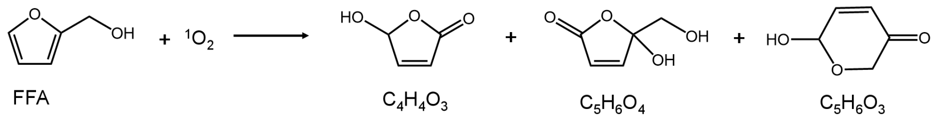

(S19)

$$A_{FFA} = \varepsilon_{FFA}\ b\ c_{FFA} \qquad (S20)$$

$$A_{product} = \varepsilon_{product}\ b\ c_{product} \qquad (S21)$$

$$c_{product} = \frac{A_{product}}{A_{FFA}} \times \frac{\varepsilon_{FFA}}{\varepsilon_{product}} \times c_{FFA} \qquad (S22)$$

Where A is the absorbance, here we express it as the chromatographic peak area, ε represents the molar adsorption coefficient, b is the optical depth through the sample,

c is the concentration of the sample. According to mass balance calculation, FFA was oxidatively converted to three classical products with a conversion rate more than 99% (Figure 2f). Hence, $^1O_2$ was considered to be the exclusive role of $^1O_2$ as the reactive species for BPA degradation in Co_3O_4-*in*-CNT/PAA system.

Supplementary Note 12: Identification of ROS in the activation of other peroxides.

The mechanism of radical generation in Fenton-like reactions is well accepted, while the production of $^1O_2$ was challenged by both kinetics and thermodynamics [24]. To explore whether this mechanism is unique for PAA, activation of other peroxides (e.g., H_2O_2, PMS and PDS) were also investigated. To further ascertain the dominant ROS for the degradation of BPA, the quenching experiments and EPR experiments are carried out. According to the previous literatures [3], the activation mechanism of hydrogen peroxide and persulfate may be a radical process dominated by $HO^\bullet$ or $SO_4^{\bullet-}$.

To verify whether $HO^\bullet$ or $SO_4^{\bullet-}$ were involved in the reaction, the corresponding scavengers, *tert*-butanol (TBA) and methanol (MeOH), respectively, were individually added to the reaction solution [27]. MeOH is utilized to scavenge both $HO^\bullet$ ($k_{HO\bullet} = 9.7 \times 10^8$ M^{-1} s^{-1}) and $SO_4^{\bullet-}$ ($k_{SO4\bullet-} = 3.2 \times 10^6$ M^{-1} s^{-1}), whereas TBA is used as scavenger for $HO^\bullet$ ($k_{HO\bullet} = 3.8$–7.6×10^8 M^{-1} s^{-1}). As shown in Supplementary Figure 15, the degradation of BPA was obviously inhibited with the presence of 100 mM TBA or MeOH in the Co_3O_4-*out*-CNT/H_2O_2 system, which indicated that $HO^\bullet$ was the primary reactive species. Furthermore, the dominant role of $HO^\bullet$ in the

Co_3O_4-*out*-CNT/PDS and Co_3O_4-*out*-CNT/PMS systems were also investigated by adding 100 mM TBA. Nevertheless, BPA degradation was not completely inhibited, thus MeOH was added to the reaction solution as a quench to elucidate e the contribution of $HO^•$ or $SO_4^{•-}$. in the degradation process. As expected, the degradation rates of BPA were significantly inhibited, indicating that radicals play a dominant role in the Co_3O_4-*out*-CNT/PDS and Co_3O_4-*out*-CNT/PMS systems. EPR analysis is another important method to verify the involvement of ROS with 5, 5-diamethyl-1-pyrroline N-oxide (DMPO) utilized as the spin trapping agent to capture the $HO^•$ or $SO_4^{•-}$ (Supplementary Figure 16). Obviously, four sharp signal adducts with the ratio of 1:2:2:1 could be observed in the Co_3O_4-*out*-CNT/H_2O_2 system, which was assigned to DMPO- $HO^•$ adduct characteristic peaks ($\alpha_H = \alpha_N =$ 14.9 G). In addition, the signals of DMPO-$SO_4^{•-}$ (α_N = 13.2 G, α_H = 9.6 G, α_H = 1.48 G and α_H = 0.78 G) are also observed in the Co_3O_4-*out*-CNT/PDS and Co_3O_4-*out*-CNT/PMS systems, which further confirmed that $HO^•$ and $SO_4^{•-}$ were responsible for BPA oxidation. Notably, sodium azide (NaN_3), as a quencher for $^1O_2$, significantly reduced the degradation of BPA in the reaction, which is attributed to the high reactivity of $^1O_2$ towards $HO^•$ or $SO_4^{•-}$ ($10^9 – 10^{10}$ M^{-1} s^{-1}) as well [4]. In addition, $^1O_2$ was not detected in the EPR spectra, further proving that $^1O_2$ was not produced in the Co_3O_4-*out*-CNT/peroxides systems. Therefore, the BPA degradation process was attributed to the radical pathway involving $HO^•$ or $SO_4^{•-}$ in the Co_3O_4-*out*-CNT/peroxides systems.

On the contrary, the degradation of BPA was not affected in the presence of 100

mM TBA or MeOH (Supplementary Figure 15) in the all Co_3O_4-*in*-CNT/peroxides systems, suggesting that the degradation of BPA could not be attributed to $HO^\bullet$ or $SO_4^{\bullet-}$. Afterwards, EPR spectra were obtained with TEMP as a trap for $^1O_2$ and DMPO as a trap for $HO^\bullet$ and $SO_4^{\bullet-}$ [28]. Signals corresponding to 2,2,6,6-tetramethylpiperidine-N-oxyl (TEMPO), which is a TEMPO–$^1O_2$ adduct with the ratio of 1:2:2:1, were observed (Supplementary Figure 16). However, no signals for either trapped DMPO- $HO^\bullet$ or DMPO-$SO_4^{\bullet-}$ were observed in all systems, which confirmed that $SO_4^{\bullet-}$ and $^\bullet OH$ did not contribute to BPA degradation. To verify the participation of $^1O_2$ during the peroxides degradation process, NaN_3 was employed, given the relatively high rate constant (2×10^9 M^{-1} s^{-1}) toward $^1O_2$ [29]. After NaN_3 was added to the solution (Supplementary Figure 15), the degradation of BPA was completely inhibited in the Co_3O_4-*in*-CNT/peroxides systems. Overall, the results suggested that $^1O_2$ play the primary role in the system.

Supplementary Note 13: The role of Co (IV) in the degradation of BPA.

Experiments were further conducted to investigate whether Co(IV) directly oxidized BPA or only acted as a crucial intermediate for $^1O_2$ production. Indeed, the high-valence metals, e.g., Co(IV), can directly oxidize organic contaminants via electron transfer, and the addition of contaminants could greatly enhance the decomposition of perxides[10, 30]. However, the PAA decomposition in the Co_3O_4-*in*-CNT/PAA system was unaffected after addition of BPA (Supplementary Figure 19), implying that Co(IV) was not directly responsible for the degradation of

BPA. In addition, chronoamperometry measurements were performed to evaluate the direct electron-transfer process between the surface of catalysts and BPA. Figure 2g shows the sudden jump in current intensity with addition of PAA, which could be attributed to the electron migration from catalysts to PAA. It is well documented that high-valent intermediates are generated through heterolytically cleavage of the peroxide bond for transition metal catalyzed systems [31]. The increase in current confirmed the process of continuous electron loss caused by the oxidation of Co(II) and Co(III) with PAA (Co(II) − $2e^-$ → Co(IV); Co(III) − e^- → Co(IV)) [32], resulting in the high-valent Co-oxo species Co(IV), i.e., through heterolytic cleavage of the O-O bond. For this situation, assuming the ensuing infusion of BPA brought about the other way of current change, implying electron transfer from BPA to Co(IV) and prompting BPA degradation [31]. However, the apparent change in current was not observed, implying the absence of direct reaction between Co(IV) and BPA.

Supplementary Note 14: Electrochemical Measurements.

Electrochemical measurements were performed using a CHI 760E electrochemical workstation equipped with standard three-electrode electrochemical cells (CHI 660E, Shanghai Chenhua Instrument Co., China). The prepared catalysts, platinum wire and Ag/AgCl electrode (saturated KCl solution) were used as the working electrode, counter electrode and reference electrode, respectively. Specifically, Na_2SO_4 (0.1 M) was utilized as the electrolyte, while the suspension catalyst dispersion consisting of 10 mg catalyst, 0.1 mL of Nafion solution and 1.0

mL of ethanol was used to prepare of the working electrodes. CV curves of different catalysts were determined at a scan rate of 50 mV•s^{-1}. EIS was recorded using a magnitude AC voltage of 5 mV over a frequency range of 10^5 - 10^{-1} Hz under -0.3 V versus Ag/AgCl.

The inductance (L) is ascribed to high frequency artifacts produced by the device [33]. Moreover, the resistor (R_0) is the resistance of the electrolyte and electrodes, while Rc and Rpn are the resistive contributions from the solid bulk matrix (Cc) and the connected pore network (Q), respectively. The Warburg impedance (W) represents the diffusion characteristic. The 450 high frequency intercept is characteristic of the Warburg impedance. Rct is the charge transfer resistance/double layer capacitance (Cpn) on the electrode surface, and the mass/redox transformation within the catalyst layer on the electrode surface.

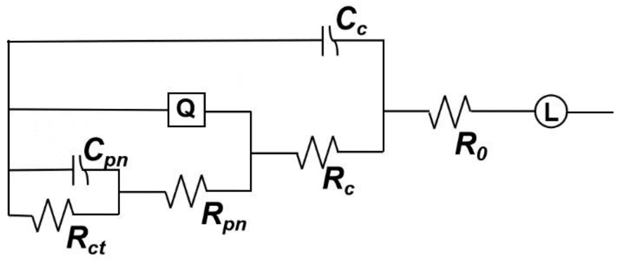

Equivalent circuit used for fitting the Nyquist plots

Supplementary Note 15: Effect of pH on BPA removal.

As shown in Supplementary Figure 25, the effect of pH on BPA degradation was investigated in the Co_3O_4-*out*-CNT system, and results showed that BPA degradation was strongly associated with the solution pH. The highest degradation rate was achieved at neutral condition with k_{obs} reached 0.069 min^{-1}. Neither acidic nor alkaline conditions were conducive to the degradation of BPA. As the pKa of PAA is 8.2, the

neutral species of PAA (PAA^0) is dominant at acidic conditions. The density functional theory (DFT) calculations showed that the optimized PAA^0 had a shorter O−O bond length than that of the anion form of PAA (PAA^-) [11], implying that PAA^0 is more difficult to be activated. The previous studies also reported that PAA^0 is much more stable in the acidic pHs [14], thus the catalytic decomposition of PAA is unfavorable in acidic conditions. Although PAA^- is easier to be activated in the alkaline condition to generate reactive species for contaminants degradation, the hydrolysis of PAA^- is accelerated at alkaline pHs, resulting in the high consumption of PAA. Besides, the isoelectric point (pH_{pzc}) of Co_3O_4-*out*-CNT was below 3.0, indicating that the catalyst was negatively charged at alkaline pHs. Thus, the heterogeneous reaction could be inhibited due to the electrostatic repulsion between PAA^- and Co_3O_4-*out*-CNT at alkaline pHs. The alkaline conditions also favored the formation of unreactive metal hydroxide complexes on catalyst surface, further reducing the catalytic activity of Co_3O_4-*out*-CNT. Accordingly, these factors together resulted in the inhibition of BPA degradation at alkaline conditions.

Supplementary Note 16: The mass transfer and chemical kinetic model.

In the heterogeneous catalysis, the produced reactive species can be localized to the catalyst surface (e.g., activated peroxide complexes, and surface-localized radicals), or diffuse from the surface in a rather limited distance (e.g., radicals, $^1O_2$, and triplet excited compounds)[34]. Specifically, the reaction is initiated by the diffusion of reactants from the bulk solution area, where PAA is activated on the catalyst surface to generate various ROS, and BPA is enriched in the solid−liquid boundary

layer region with near catalyst surface. Afterwards, the produced ROS left catalyst surface to react with BPA in the solid−liquid boundary layer region. Hence, the diffusion of oxidants toward the bulk solution and the diffusion of BPA toward the interfacial region are crucial to realize the rapid oxidation. Though it is difficult to precisely portray the diffusion of the reactants in a real system, the mass transfer and chemical kinetic model models were developed for better understanding the distributions on or near the solid−liquid interface. The diffusion of reactive oxidants is described by the modified Fick's second law. Of these, it is worth noting that the diffusion model is proposed for a neutral oxidant assuming no interaction with the surface.

$$\frac{\partial c_{\mathrm{ox}}}{\partial t} = D\frac{\partial^2 c_{\mathrm{ox}}}{\partial x^2} - v_{\mathrm{consumed}} = D\frac{\partial^2 c_{\mathrm{ox}}}{\partial x^2} - \sum_i k_i c_{\mathrm{ox}}[S_{i,x}] \qquad \text{(S23)}$$

Where Cox is the concentration of reactive oxidants (M); D is the diffusion coefficient (m^2 s^{-1}); t is the diffusion time (s); x is the diffusion distance (m); k_i is the second-order rate constant (M^{-1} s^{-1}); $v_{consumed}$ is the rate at which the reactive oxidant is consumed by reaction with the substrate in solution (s^{-1}); [$S_{i,x}$] is the species concentration at x from the surface (M). For example, when the target contaminant and the catalyst surface have opposite charges, then the target contaminant will be concentrated near the catalyst surface. In addition, the attractive or repulsive force of the catalyst surface to the charged oxidant should also be considered. Then the distribution of counter ions and co-charged ions in the diffusion layer conforms to the Boltzmann distribution:

$$n_x = n_0 \exp\left(\frac{ze\varphi_x}{k_B T}\right) \qquad \text{(S24)}$$

where n_0 and n_x are the number of the ions in the bulk solution and at x, respectively; z delegates the charge of ions; φ_x is the potential at x (V); k_B represents the Boltzmann constant (1.38×10^{-23} J K^{-1}); T is the absolute temperature (K). Based on the predictions of the electric double layer theory proposed in the Helmholtz model, we know that the distribution of ions depends on the distribution of electric potential. In other words, electrostatic interactions and van der Waals forces cause the charged surface of the colloid to be balanced by a compact layer of ions on the solution side. Therefore, the Gouy-Chapman model was established and further modified by Stern, which considers the specific adsorption and intrinsic properties of counterions, such as thermal motion, ionic radius, and solvation of ions. The Gouy-Chapman-Stern model contains compact layers close to the surface, which called as Stern layers, and diffusion layers out of the Stern plane. The compact stern layer has a thickness of less then10 Å and is charge-free, thus resulting in a constant potential gradient. However, in the diffusion layer, the potential gradient can be described by the Poisson-Boltzmann equation:

$$\frac{d\varphi}{dx} = -\left(\frac{8k_BTN_Ac}{\varepsilon\varepsilon_0}\right)^{1/2} \sinh\left(\frac{ze\varphi}{2k_BT}\right) \quad \text{(S25)}$$

where φ is the potential (V); x is the distance from the Stern plane (m); N_A is Avogadro constant (6.02×10^{23}); c is the concentration of ions (M); ε_0 and ε are the free space permittivity and the relative permittivity of the solution (F m^{-1}), respectively. Therefore, the above equation (eq S26) can be rearranged and integrated as follows:

$$\tanh\left(\frac{ze\varphi_x}{4k_BT}\right) = \tanh\left(\frac{ze\varphi_0}{4k_BT}\right) \exp\left(-\left(\frac{e^2N_AI}{\varepsilon\varepsilon_0k_BT}\right)^{1/2} x\right) = \tanh\left(\frac{ze\varphi_0}{4k_BT}\right) \exp\left(-\kappa x\right) \quad \text{(S26)}$$

Where I represent the ionic strength; κ is the Debye length and is also considered to be

the thickness of the diffusion layer (m). Afterwards, integrating eq S24 and eq S26, the ion distribution in the diffusion layer can be obtained as eq S27:

$$\tanh\left(\frac{1}{4}\ln\left(\frac{c_x}{c_0}\right)\right) = \tanh\left(\frac{ze\varphi_0}{4k_BT}\right)\exp\left(-\kappa x\right) \qquad \text{(S27)}$$

Kinetic model: The kinetic model based on first-principles was developed using the software package MATLAB_R2020 that were capable of describing the concentrations of all species present as a function of time and process conditions, and the kinetic model refers to our previous work [1]. Briefly, the first step was to identify the possible elementary reactions that involved the transformation of reactive species and the degradation of pollutant. The second step was to determine the rarely reported second-order rate constants for the reactions of reactive species with BPA. The unknown second-order rate constant is determined by using genetic algorithm (GA) to minimize the objective function (OF). OF in eq S28 reflects whether the simulation results fit the experimental data well:

$$OF = \sqrt{\frac{1}{N-1}\sum_{i=1}^{N}\left[\frac{C_{exp}-C_{cal}}{C_{exp}}\right]^2} \qquad \text{(S28)}$$

where N is the number of experimental data points, C_{exp} and C_{cal} are the experimental and calculated concentration of parent pollutant at each time point, respectively. Among them, the number of populations in the algorithm is set as 8, the number of optimizing steps is set to 3000, and the quad-core parallel operation is adopted.

The third step is to predict the concentration distribution of parent pollutant with reaction time. In order to describe the degradation of parent pollutant in ultrapure water, a set of stiff ordinary differential equations (ODEs) was established for the mass balance equations for all species in the batch reactor due to the wide range of

rate constants. We used Gear's method based on the backward differentiation formula to solve the stiff ODEs. Solving stiff ODEs are computational cost expensive and results are extremely unstable [35]. Therefore, we use the Gear's method to solve the stiff ODEs, which is based on the backward differential formula and is one of the most effective and stable methods to solve the stiff ODEs [36]. Finally, the model was validated by comparing the modeling results to the experimental data.

We analyzed the sensitivity of experimental parameters to validate the model. Total S values (eq S29-S31) were used to evaluate the importance of individual reactions for the evolution of major species and the overall kinetics of Fenton-like system. The larger the Total S value, the more important the reaction is to the whole simulation result (Supplementary Table 11-12).

$$s_j = \sum_1^n \left(\frac{\frac{C_i^{k_j^\alpha}}{C_i^{exp}} + \frac{C_i^{k_j^\beta}}{C_i^{exp}}}{2}\right) \quad \text{(S29)}$$

$$S_j = \frac{s_j - \min_m s}{\max_m s - \min_m s} \quad \text{(S30)}$$

$$Total\,S = \sum_1^m S_j \quad \text{(S31)}$$

Where s_j, S_j and $Total\,S$ are the sensitivity index, standardized sensitivity index and total standardized sensitivity index, respectively; m is the number of reactions; n is the number of species; j is raction j and range from 1 to m; α is upper limit of parameter change, 10% or 20%; β is lower limit of parameter change, -10% or -20%; $C_i^{k_j^\alpha}$ is the modeled value of contaminants concentration timepoint i, with a 10% or 20% increase on k_j; $C_i^{k_j^\beta}$ is the modeled value of contaminants concentration timepoint i, with a 10% or 20% decrease on k_j.

Supplementary Note 17: The calculation Debye-Hückel distance.

The description of charged surfaces in aqueous environments involves the extension of the electrostatic bilayer to the solution in the diffuse part, which starts from the outer Helmholtz plane. The electric field (E, N C^{-1}) for linear, isotropic and homogeneous materials can be described with the Poisson equation as eq S32:

$$\nabla E = \nabla(-\nabla\psi) = -\nabla^2\psi = \frac{\rho}{\varepsilon\varepsilon_0} \qquad \text{(S32)}$$

where ψ refers to the surface potential of the distance to the outer Helmholtz plane (V); ρ is the charge density, (C m^{-3}); ε and ε_0 are the dielectric permittivity of the medium and the vacuum permittivity, respectively. Outside the outer Helmholtz plane, there is a bunch of oppositely charged ions. The ion concentration distribution is described by the Boltzmann distribution, and the charge density can be expressed as:

$$\rho = \sum_i z_i e n_i = \sum_i z_i e n_i^{\infty} \exp\left(-\frac{z_i e\psi}{kT}\right) \qquad \text{(S33)}$$

where z_i represents the valence of the "i" ion; e is electronic charge (C); n_i is the total number of "i" ions near interface, dimensionless; $ni\infty$ is the total number of "i" ions in bulk phase, dimensionless; k is the Boltzmann constant, (J K^{-1}); T is the thermodynamic temperature (K). Considering the gradient of charges from OHP, the potential ψ as a function of distance to the surface (L, m) can be expressed by eq S34, taking L as the direction perpendicular to the solid solution interface. Consider the outer Helmholtz plane charge gradient and perpendicular to the direction of the solid solution interface for L, thus the function of the potential ψ and the surface distance (L, m) can be expressed through eq S34:

$$\frac{d^2\psi}{dL^2} = -\frac{e}{\varepsilon\varepsilon_0}\sum_i z_i n_i^{\infty} \exp\left(-\frac{z_i e\psi}{kT}\right) \qquad \text{(S34)}$$

Considering the electrical neutrality in bulk solution ($\sum i\ zien\frac{\infty}{\mathrm{i}}$ = 0), the exponential

term is approximated, and eq S34 is simplified as follow:

$$\frac{d^2\psi}{dL^2} = \frac{1}{\varepsilon\varepsilon_0 kT}\sum_i z_i^2 n_i^\infty e^2\psi = \kappa^2\psi \qquad (S35)$$

where κ is defined as the Debye-Hückel parameter (m^{-1}). We assumed that L → d, ψ = ψd (d is the thickness of Stern layer) and L → ∞, ψ = 0, the Debye-Hückel length can be calculated by

$$\kappa^{-1} = \left[\frac{N_A e^2}{\varepsilon\varepsilon_0 kT}\sum_i z_i^2 c_i^\infty\right]^{-\frac{1}{2}} \qquad (S36)$$

where N_A represents the Avogadro constant, dimensionless; $c\frac{\infty}{i}$ is the concentration of ions of species "*i*", M. Hence, the value κ^{-1} for experiments performed at normal temperature (about 298.15 K) can be calculated.

Supplementary Note 18: Lifetime estimation of reactive oxygen species.

In the bulk phase, the decomposition of ROS can be expressed by eq S37, as follow [37]:

$$\frac{dC_{ROS}}{dt} = (\textstyle\sum_{i=1}^{n} k_{ROS/i} \cdot C_i) \cdot C_{ROS} \qquad (S37)$$

where C_{ROS} is the concentration of $^1O_2$ or R-O•, M; C_i is the concentration of reactant "*i*" consuming ROS, M; $k_{ROS/i}$ is the second-order rate constant for ROS in reaction with probe "i", M^{-1} s^{-1}. At first, we assumed that the consumption of ROS was primarily completed by the BPA, H_2O, and ROS self-quenching. Hence, the kinetics of ROS decomposition could be expressed by eq S38:

$$\frac{dC_{ROS}}{dt} =- (k_{ROS/BPA} \cdot C_{BPA} + k_{ROS/H_2O} \cdot C_{H_2O} + k_{ROS/ROS} \cdot C_{ROS}) \cdot C_{ROS} \qquad (S38)$$

When 99.9% decay of ROS occurs, we define it as the lifetime of ROS (τ_{ROS}, s), then this value can be represented as:

$$\tau_{ROS} \approx \frac{-ln(0.001)}{k_{obs,ROS}} \qquad (S39)$$

Consequently, the lifetimes of $^1O_2$ and R-O$^\bullet$ in the Co_3O_4-*in*-CNT and Co_3O_4-*out*-CNT were predicted with $\tau_{^1O_2}$ = 4.0 μs and $\tau_{R\text{-}O\bullet}$ = 134 μs, respectively.

In addition, the diffusion distance scale of ROS in the aqueous phase (λ_L) would be calculated by:

$$\lambda_L = 2\sqrt{D_{ROS} \times \tau_{ROS}} \qquad (S40)$$

where D_{ROS} is the diffusion coefficient 2.3 $\times 10^{-9}$ m^2 s^{-1} [37]. Therefore, the diffusion distance of $^1O_2$ and R-O$^\bullet$ (λ_L) were calculated to be~192 nm and ~1110 nm, respectively.

Supplementary Note 19: Effect of water matrices on BPA degradation.

Water matrices, e.g., anions and natural organic matter (NOM), are known to decrease Fenton-like reaction efficiencies due to the radical scavenging, and competitive adsorption onto nanomaterials surfaces. The results showed that BPA degradation was almost unaffected by the coexisting ions in the Co_3O_4-*in*-CNT/PAA system (Supplementary Figure 39a). Indeed, $^1O_2$, a selective oxidant, is less affected by the water matrices, e.g., ions than the radicals. Additionally, the nonradical oxidation originated from spatial nanoconfinement can overcome the known challenges occurred in the radical-based oxidation. For instance, the formation of toxic halogenated organic (DBPs) and inorganic byproducts (e.g., chlorate) is a significant concern in radical-based oxidation processes due to the reaction of R-O$^\bullet$ with halogen ions (e.g., Cl^-) [15]. As expected, the BPA degradation was distinctly inhibited by NOM in the unconfined system owing to the shield of the active sites of

Co_3O_4 NPs by NOM. Additionally, NOM could compete with BPA for R−O$^•$ due to the high reactivity of R−O$^•$ with NOM. Nevertheless, the BPA degradation was minimally affected by NOM in the confined system. Although NOM also exhibits high reactivity to $^1O_2$, the interference by NOM can be largely avoided under spatial nanoconfinement. Specially, the majority of macromolecular compounds and colloidal water components (e.g., NOM and soluble microbial products) could be excluded from Co_3O_4-*in*-CNT by size exclusion, thus the internal space can still serve as catalytic sites in the presence of NOM (Figure 5b) [38, 39].

The impacts of common water matrices including inorganic anions and cations, and natural organic matter (NOM) were further examined in the Co_3O_4-*out*-CNT/PAA system. Results showed that the BPA degradation was less affected by inorganic cations, NO_3^- and SO_4^{2-} (Supplementary Figure 39b), but was significantly inhibited by Cl^- and HCO_3^- (Supplementary Figure 40). Cl^- is also a common influencing factor in AOPs as it can rapidly react with reactive radicals to produce a sequence of reactive chlorine-containing species, such as $Cl^•$, $ClOH^{•-}$ and $Cl_2^{•-}$ [40].The impacts of Cl^- on the degradation of different organic pollutants are quite different due to the high-selective oxidation ability of the generated reactive chlorine-containing species. R−O$^•$ (i.e., $CH_3C(O)OO^•$) has been reported to react with Cl^- to generate $Cl^•$ [41], thus the effect of Cl^- on the degradation of BPA by Co_3O_4-*out*-CNT/PAA in the concentration range of 0−100 mM was investigated. The result indicates that the addition of Cl^- played a significant role in BPA degradation (Supplementary Figure 40). The formed reactive chlorine species might account for the degradation of BPA, since the degradation of

BPA is not completely inhibited when the concentration of Cl^- increased to 100 mM. Notably, the reactive chlorine species are prone to react with coexisting substrates in water to generate chlorine-based disinfection by-products, which seriously endanger ecosystems and human health.

As a common scavenger of reactive radicals, the influence of HCO_3^- on the degradation of BPA in the in the Co_3O_4-*out*-CNT/PAA system was also evaluated (Supplementary Figure 40). As the reaction pH kept at around 7.0 during the reaction, HCO_3^- should be the dominant species in the system as the pK_{a1} = 6.37. Although HCO_3^- is a common scavenger of $HO^•$ and $SO_4^{•-}$, the reactivity of HCO_3^- with $CH_3C(O)OO^•$ is largely uncertain. Previous studies reported that the activity of $CH_3C(O)OO^•$ towards HCO_3^- was lower than that of $HO^•$ ($k_{HO•/HCO3-} = 8.5 \times 10^6$ M^{-1} s^{-1}) [16]. Therefore, the consumption of the formed R−$O^•$ through the reaction between HCO_3^- and R−$O^•$ would be the primary reason for the inhibition of BPA degradation in the Co_3O_4-*out*-CNT/PAA process. In contrast, $^1O_2$ is more resistant to environmental interferences, such as the presence of Cl^- and HCO_3^-.

Supplementary Note 20: Degradation pathway and toxicity analysis.

The degradation pathway and toxicity of BPA were further investigated in the Co_3O_4-*in*-CNT/PAA system. The reactive sites of BPA were predicted by the highest occupied molecular orbital (HOMO) and condensed Fukui functions (CFF) of BPA. The results showed that the electrophilic attack sites were primarily located on the benzene ring region, and the sites with higher f^- values (O30, O32, C1, C6, C14 and C15) are potential electrophilic attack sites (Supplementary Figure S27). The

transformation products (TPs) of BPA were analyzed based on the corresponding fragmentation patterns in the MS/MS spectra (Supplementary Figures 28-36). As shown in Supplementary Figure 37, BPA is degraded mainly through hydroxylation, ring opening and decarboxylation pathways. Specifically, BPA was oxidized to the hydroxylation product **TP244**, and the phenolic substituents were oxidized to the benzoquinone product **TP256** via one-electron transfer. Subsequently, the benzoquinone structure of TP256 is further oxidized to carboxylic acid products (**TP324**) by ring-opening reaction. In addition, the C1 or C15 with high f^- values suffered from an electrophilic attack by $^1O_2$, causing benzene ring cleavage and dicarboxylic acid byproducts formation (**TP266** and **TP194**). Afterwards, the reaction products were further oxidized to **TP194** and **TP180** via decarboxylation and demethylation. Finally, these intermediates of BPA degradation could further react with $^1O_2$ and convert to CO_2 and H_2O.

The aquatic toxicity of TPs was estimated by QSAR analysis using the ECOSAR system. The results show that all TPs exhibit much lower toxicity than BPA, i.e., mainly due to the cleavage of the aromatic ring, which are the major biotoxic groups (Supplementary Table 13). The toxicity of each product during the reaction was measured by the above method, and then we determine the acute toxicity of the solution after treatment by measuring the inhibition rate of luminescence against *Vibrio fischeri* bacteria. As shown in Supplementary Figure 38, the luminescence inhibition obviously reduced to 11.5% in the first 15 min, and then reduced 1.5% when the reaction time was further increased to 60 min, which was attributed to the

transformation of intermediates to other less toxic byproducts along the reaction. Overall, $^1O_2$ not only selectively removed water contaminants but also significantly decreased their toxicity.

Supplementary Note 21: Selective oxidation strategies for decontaminants.

Fenton-like oxidation processes are frequently employed to degrade organic pollutants for water purification. However, the quenching of radicals in Fenton-like reactions by various inorganic anions or organic compounds limits their value in industrial applications. The need to address this challenge has motivated intensive research on selective oxidation technologies via nonradical process. Hence, we proposed two pathways to promote selective oxidation for rapid decontamination: tuning the reactive species to enhance selectivity and enhancing mass transfer process to improve kinetics. Specifically, nonradical process (e.g., $^1O_2$ and high-valent metals) typically exhibit high selectivity toward electron-rich organic substances, and maintain excellent efficiency in complicated water matrices in a wide pH working window, despite the presence of inorganic anions and NOM. In addition, the selective oxidation strategy is to enhance the mass transfer process and improve the kinetics by altering the selectivity of the catalyst. For example, selective contacts between catalysts and contaminants (including size exclusion, surface charge, hydrophobicity). Given the complex components of wastewater, combining various methods for selective oxidation in actual wastewater treatment prove to be promising approach.

Overall, nonradical pathways (e.g., $^1O_2$) has several advantages, including: a) making full utilization of oxidants' oxidizing capacities; b) avoiding the

self-quenching of radicals produced during the reaction; and c) minimizing interference from organic and inorganic compounds in the real water environment. In addition to the abovementioned advantages, the selective (mild) oxidation capacity inevitably has some drawbacks. Specifically, nonradicals with mild oxidation capabilities have comparatively slow degradation rate and low mineralization rates for the target organic contaminants. Hence, appropriate strategies should be employed to maximize the function of nonradical oxidation mechanism in practical applications. Firstly, the oxidation of refractory pollutants has been evidenced as a good method to improve the biodegradability, implying that oxidation would be integrated with the subsequent biotreatment procedure. In comparison with other Fenton-like systems, activated PAA processes would be more appropriate to associate with biotreatment process as the catalytic decomposition of PAA can produce numerous fine carbon sources including methanol, acetic acid and formaldehyde. Hence, combining PAA-based oxidation process and biotreatment process would contain bright potential in the treatment of contaminated waters, especially when the waters are lack of carbon sources. Secondly, the $^1O_2$ oxidation process can be employed as pretreatment technique in the wastewater treatment, because $^1O_2$ can successfully oxidize organic materials into intermediate products with simpler or harmless structures. In addition, the $^1O_2$ oxidation is inert towards Cl^- compared with the radical oxidation process, thus the nonradical based oxidation process avoid the secondary pollution of harmful by-products (e.g., ClO_3^- and BrO_3^-).

Supplementary Table 1. HPLC method parameters for contaminants detection.

Chemical	Mobile Phase	Injection volume (μL)	Wavelength (nm) [a]	Flow Rate (mL/min)
BPA	methanol/water (70:30, v/v%)	100	280	1
SMX	acetonitrile/water (0.4% CH_3COOH) (29:71 v/v)	100	275	1
4-CP	acetonitrile/water (0.1% CH_3COOH) (38:62 v/v)	100	280	1
IMD	methanol/water (0.1% CH_3COOH) (55:45 v/v)	100	270	1
EE2	Acetonitrile/water (0.1% CH_3COOH) (70:30 v/v)	100	λ_{ex} = 217 nm; λ_{em} = 300 nm[b]	1
BA	acetonitrile/water (0.1% CH_3COOH) (50:50 v/v)	20	230	1
NB	acetonitrile/water (0.1% phosphate) (50:50 v/v)	100	265	1
CBZ	acetonitrile/water (0.1% CH_3COOH) (50:50 v/v)	100	285	1
ATZ	methanol/water (0.1% CH_3COOH) (70:30 v/v)	100	221	1

[a] The detection wavelength was chosen according to the maximum absorbance in UV-vis spectrum of individual compound.

[b] Fluorescence detector was set with excitation wavelength of λ_{ex} = 217 nm and emission wavelength of λ_{em} = 300 nm.

Supplementary Table 2. Specific surface area of CNTs, Co_3O_4-*out*-CNT and Co_3O_4-*in*-CNT.

Catalysts	$S_{BET}(N_2)$ (m^2 g^{-1})	Total pore volume (cm^3 g^{-1})	Pore diameter (nm)
CNTs	126.7	0.6187	19.025
Co_3O_4-*out*-CNT	164.4	0.5645	3.063
Co_3O_4-*in*-CNT	180.8	0.5215	2.879

Supplementary Table 3. **The catalytic performance comparison of previously reported Fenton-like catalysts for PMS, H_2O_2 and PAA activation.**

Catalyst (g L^{-1})	$PMS^a/H_2O_2^b/PAA^c$ (g L^{-1})	BPA (μmol L^{-1})	Removal Efficiency (%)	k_{obs} (min^{-1})	k-value [d]	Ref.
DPA-hematite (0.5)	2.0[a]	65.7	100 (120 min)	0.039	0.039	42
$CuFe_2O_4$-Fe_2O_3 (0.2)	0.36[a]	21.9	100 (10 min)	0.620	8.611	43
$Mn_{1.8}Fe_{1.2}O_4$ (0.1)	0.2[a]	43.8	95 (30 min)	0.102	5.1	44
Fe^{3+}-g-C_3N_4 (0.1)	0.3[a]	100.8	100 (15 min)	0.302	10.067	44
$Fe_{0.8}Co_{2.2}O_4$ (0.1)	0.2[a]	87.6	95 (60 min)	0.049	2.45	45
Fe_3Co_7@C (0.1)	0.2[a]	87.6	95 (30 min)	0.132	6.6	44
$Fe_1Mn_5Co_4$-N@C (0.1)	0.2[a]	87.6	100 (10 min)	0.480	24	46
Ag/AgCl/Fh (1.0)	0.18[b]	131.4	100 (60 min)	0.050	0.278	47
Cn-Cu(II)-$CuAlO_2$ (1.0)	0.18[b]	109.6	98 (120 min)	0.030	0.167	48
Ag/AgCl/Fe-S (1.0)	0.1[b]	43.8	100 (120 min)	0.030	0.3	47
Cu-Al_2O_3 (1.0)	0.2[b]	87.6	87 (180 min)	0.010	0.05	48
Cu-doped $AlPO_4$ (1.0)	0.18[b]	109.6	92 (180 min)	0.010	0.056	46
S modified Fe_2O_3 (0.2)	0.04[b]	192.7	100 (20 min)	0.314	39.25	46
Co@N−C-KNO_3 (0.025)	0.6[a]	219	100 (10 min)	0.730	48.67	32
Fe-SAC (0.2)	0.4[a]	109.6	88 (15 min)	0.104	1.30	49
Co-SAC (0.2)	0.4[a]	109.6	79 (15 min)	0.0832	1.04	49
Mn-SAC (0.2)	0.4[a]	109.6	70 (15 min)	0.0624	0.78	49
Ni-SAC (0.2)	0.4[a]	109.6	62 (15 min)	0.052	0.65	49
Cu-SAC (0.2)	0.4[a]	109.6	56 (15 min)	0.046	0.58	49
FeCo-NC-1 (0.1)	0.2[a]	87.6	100 (5 min)	1.179	58.95	46

FeCo-NC-2 (0.1)	0.2[a]	87.6	100 (4 min)	1.252	62.6	[46]
FeCo-NC-3 (0.1)	0.2[a]	87.6	95 (6 min)	0.465	23.25	[46]
2D Fe_3O_4 nanosheets (0.2)	0.5[a]	87.6	93.2 (10 min)	0.222	2.22	[50]
$Fe_1Mn_5Co_{4\text{-}N}$@C (0.1)	0.2[a]	87.6	100 (10 min)	0.48	24	[51]
yolk−shell Co/C (0.15)	0.1[a]	87.6	98 (15 min)	0.5	33.33	[52]
Co_3O_4-*out*-CNT (0.1)	0.0152[c]	100	52.6 (15 min)	0.049	32.30	This work
Co_3O_4-*in*-CNT (0.1)	0.0152[c]	100	95.8 (15 min)	0.188	**116.12**	This work

[d]: The normalized rate constant (k-value) was calculated through dividing the reaction rate of pollutant degradation by the catalyst and oxidant concentration.

Supplementary Table 4. Physical-chemical properties and reactivities of the main oxidants.

	Hydrogen peroxide (H_2O_2)	Peroxymonosulfate (PMS) [a]	Peroxydisulfate (PDS) [a]	Peracetic acid (PAA)
Standard reduction potential (E^0)	1.8 V_{NHE}	1.82 V_{NHE}	2.08 V_{NHE}	1.96 V_{NHE}
Peroxide bond dissociation energy	213 kJ mol^{-1}	377 kJ mol^{-1}	92 kJ mol^{-1}	159 kJ mol^{-1}
Molar absorption coefficient	18.6 $M^{-1}cm^{-1}$	19.1 $M^{-1}cm^{-1}$	27.5 $M^{-1}cm^{-1}$	8.0 $M^{-1}cm^{-1}$
Acid dissociation constant (pK_a)	10.5	9.3	-3.5	8.2
Preferred activation method	Energy-transfer-based activation (e.g., thermolusis, photolysis)	Electron-transfer-based activation (e.g., catalysis with transition metals and nanocarbons)	Energy-transfer-based activation (e.g., thermolusis, photolysis)	Electron-transfer-based activation (e.g., catalysis with transition metals)

[a] Data refer to previous literature [3].

Supplementary Table 5. EPR Parameters of DIPPMPO spin trapping for the radicals in the Co_3O_4-*out*-CNT/PAA system.

Radicals	α_N,G		α_H,G		α_P,G	
	This study	Literatures [53, 54, 55, 56, 57]	This study	Literatures[16]	This study	Literatures[7]
$HO^{\bullet}$	13.6	14.0, 14.1	13.4	13.2, 13.5, 13.4	46.6	46.5, 46.5, 46.8
$CH_3C(O)O^{\bullet}$	10.2	10.2, 10.3	13.6	13.7, 13.9	50.3	50.9
$CH_3^{\bullet}$	14.5	14.4, 14.8, 15.1	22.3	22.8, 22.3, 22.6	47.1	45.5, 47.1,
$CH_3OO^{\bullet}$	13.8	14.1, 14.0	11.2	11.0, 11.1	46.5	46.7, 46.8

Reaction conditions: $[PAA]_0$ = 200 μM, $[Co_3O_4\text{-}out\text{-}CNT]_0$ = 0.1 gL^{-1}, $[DIPPMPO]_0$ = 100 mM, pH = 7.0.

Supplementary Table 6. The O-O and C-O bond lengths of PAA adsorbed on the catalysts.

Structures	$l_{O\text{-}O}$ (Å)	$l_{C\text{-}O}$ (Å)
PAA	1.458	1.364
CNTs	1.459	1.365
Co_3O_4	1.455	1.362
Co_3O_4-*out*-CNT	1.462	1.368
Co_3O_4-*in*-CNT	1.478	1.372

Supplementary Table 7. HOMO and LUMO values of catalysts with different structures.

Structures	HOMO (eV)	LUMO(eV)	E_{HOMO}-E_{LUMO}
CNTs	-5.731	-5.145	-0.586
Co_3O_4	-4.956	-4.421	-0.535
Co_3O_4-*out*-CNT	-5.045	-4.782	-0.263
Co_3O_4-*in*-CNT	-5.266	-5.195	-0.071

Supplementary Table 8. Cartesian coordination of BPA for DFT calculations.

Center number	Atom number	Coordinates (Angstroms) X	Y	Z
1	C	-0.107815	1.21103	0.648085
2	C	0.969018	1.542951	-0.185089
3	C	0.895173	2.67472	-1.008234
4	C	-0.255505	3.474571	-0.998201
5	C	-1.332339	3.142651	-0.165026
6	C	-1.258494	2.010881	0.658117
7	H	-0.051433	0.346899	1.276575
8	H	1.847588	0.932249	-0.192746
9	H	-0.311888	4.338703	-1.626689
10	H	-2.210908	3.753353	-0.157367
11	C	3.044691	3.970257	-1.167684
12	C	4.096566	3.427955	-0.417091
13	C	4.97579	4.274971	0.270985
14	C	4.80314	5.66429	0.208468
15	C	3.751267	6.206592	-0.542127
16	C	2.872042	5.359575	-1.230203
17	H	4.228387	2.367179	-0.369356
18	H	5.778919	3.860912	0.844082
19	H	3.619445	7.267367	-0.589859
20	H	2.068911	5.773634	-1.803296
21	C	2.078507	3.039469	-1.923808
22	C	2.821419	1.755818	-2.338444
23	H	2.15011	1.109102	-2.863804
24	H	3.643607	2.009248	-2.97459
25	H	3.186721	1.257218	-1.46503
26	C	1.552747	3.757081	-3.180872

27	H	2.374934	4.010511	-3.817018
28	H	0.881438	3.110365	-3.706231
29	H	1.036568	4.648968	-2.892781
30	O	5.700307	6.528592	0.910589
31	H	6.437972	6.75597	0.339844
32	O	-2.357303	1.672187	1.508296
33	H	-2.959601	1.091956	1.036948

Supplementary Table 9. Cartesian coordination of PAA for DFT calculations.

Center	Atom	Coordinates (Angstroms)		
number	number	X	Y	Z
1	C	-0.814314	0.534781	0.005125
2	H	-0.426275	-0.456074	0.117073
3	H	-0.222256	1.071399	-0.706499
4	H	-1.826441	0.484757	-0.338381
5	C	-0.768214	1.26054	1.362604
6	O	-0.622131	2.50976	1.403563
7	O	-0.89141	0.514889	2.576576
8	O	0.306118	0.13981	2.98602
9	H	0.223413	-0.360767	3.800994

Supplementary Table 10. Experimental parameters calculated by the mass transfer and kinetic model.

Simulation parameters	Co_3O_4-*in*-CNT	Co_3O_4-*out*-CNT
PAA adsorption (s^{-1})	0.003	0.001
PAA desorption (s^{-1})	1×10^8	1.24×10^5
BPA adsorption (s^{-1})	0.019	0.03
BPA desorption (s^{-1})	0.009	0.006
ROS production rate (s^{-1})	1.9×10^9	2.6×10^5
BPA degradation rate (M^{-1} s^{-1})	2.15×10^8	4.35×10^9
Average concentration of reactive species on the solid-liquid interface (M)	1.50×10^{-12}	5.41×10^{-13}
Average concentration of reactive species on catalyst surface (M)	8.71×10^{-12}	3.03×10^{-12}
Reactive species lifetime (μs)	4	134
Diffusion distance (m) of the reactive species	1.92×10^{-7}	1.1×10^{-6}

Supplementary Table 11. Calculated sensitivity measure *S* for BPA and PAA in water and surface in the Co_3O_4-*in*-CNT/PAA system.

Simulation parameters	Parameter change	Water		Surface		
		PAA	BPA	PAA	BPA	Total
PAA adsorption (s^{-1})	10%	-0.807	-1.139	0.111	-1.376	-3.211
	-10%	0.904	1.287	-0.185	1.567	3.574
	20%	-0.764	-1.075	0.082	-1.294	-3.050
	-20%	0.960	1.372	-0.231	1.678	3.778
BPA adsorption (s^{-1})	10%	0.000	-0.754	0.000	0.354	-0.400
	-10%	0.000	0.883	0.000	-0.412	0.471
	20%	0.000	-0.703	0.000	0.331	-0.371
	-20%	0.000	0.965	0.000	-0.449	0.516
ROS production rate (s^{-1})	10%	-0.275	1.692	-0.869	2.181	2.729
	-10%	0.326	-1.994	1.045	-2.574	-3.197
	20%	-0.255	1.573	-0.802	2.026	2.543
	-20%	0.359	-2.189	1.161	-2.828	-3.496
BPA degradation rate (M^{-1} s^{-1})	10%	0.000	-0.113	0.000	-0.141	-0.253
	-10%	0.000	0.114	0.000	0.143	0.257
	20%	0.000	-0.112	0.000	-0.140	-0.252
	-20%	0.000	0.115	0.000	0.144	0.259
PAA desorption (s^{-1})	10%	0.294	-1.798	-0.053	-2.321	-3.879
	-10%	-0.302	1.865	0.048	2.403	4.013
	20%	0.289	-1.767	-0.056	-2.281	-3.814
	-20%	-0.307	1.900	0.045	2.446	4.083
BPA desorption (s^{-1})	10%	0.000	0.604	0.000	-0.271	0.332
	-10%	0.000	-0.640	0.000	0.288	-0.352
	20%	0.000	0.587	0.000	-0.264	0.323
	-20%	0.000	-0.660	0.000	0.297	-0.363

Supplementary Table 12. Calculated sensitivity measure *S* for BPA and PAA in water and surface in the Co_3O_4-*out*-CNT/PAA system.

Simulation parameters	Parameter change	Water		Surface		
		PAA	BPA	PAA	BPA	Total
PAA adsorption (s^{-1})	10%	-0.300	-0.291	0.670	-0.739	-0.660
	-10%	0.312	0.313	-0.719	0.802	0.708
	20%	-0.294	-0.281	0.647	-0.710	-0.638
	-20%	0.319	0.316	-0.745	0.817	0.708
BPA adsorption (s^{-1})	10%	0.000	-0.418	0.000	0.897	0.479
	-10%	0.000	0.455	0.000	-0.960	-0.505
	20%	0.000	-0.533	0.000	0.544	0.011
	-20%	0.000	0.476	0.000	-0.995	-0.519
ROS production rate (s^{-1})	10%	-0.091	0.155	-0.722	0.429	-0.229
	-10%	0.106	-0.181	0.843	-0.501	0.267
	20%	-0.085	0.145	-0.674	0.400	-0.213
	-20%	0.115	-0.198	0.920	-0.546	0.291
BPA degradation rate (M^{-1} s^{-1})	10%	0.000	-0.084	0.000	-0.220	-0.304
	-10%	0.000	0.089	0.000	0.235	0.324
	20%	0.000	-0.171	0.000	-0.408	-0.579
	-20%	0.000	0.092	0.000	0.243	0.335
PAA desorption (s^{-1})	10%	0.096	-0.163	-0.218	-0.452	-0.737
	-10%	-0.101	0.171	0.227	0.473	0.770
	20%	0.093	-0.160	-0.213	-0.442	-0.722
	-20%	-0.103	0.176	0.232	0.484	0.788
BPA desorption (s^{-1})	10%	0.000	0.212	0.000	-0.447	-0.235
	-10%	0.000	-0.233	0.000	0.496	0.263
	20%	0.000	0.203	0.000	-0.426	-0.223
	-20%	0.000	-0.245	0.000	0.525	0.280

Supplementary Table 13. Estimated acute and chronic toxicity for fish, daphnid and green algae of BPA and transformation products by ECOSAR.

Compound	Acute toxicity [a]			Chronic toxicity [a]		
	Fish (96-h LC_{50})	Daphnid (48-h LC_{50})	Green algae (96-h EC_{50})	Fish (ChV)	Daphnid (ChV)	Green algae (ChV)
BPA	1.28	5.24	1.33	0.55	1.77	0.227
**TP224**	2.26	13.1	2.07	1.22	4.57	0.329
**TP256**	126	73.4	60.1	12.7	7.63	16.6
**TP324**	1.10E+4	5.85E+3	5.35E+3	995	478	763
**TP250**	113	66.9	10.2	12.0	8.27	25.5
**TP266**	297	136	26.3	29.8	15.2	57.5
**TP194**	90.7	53.3	8.19	9.62	6.57	20.4
**TP180**	327	131	28.6	31.8	13.8	58.1
**TP114**	1.34E+4	3.37E+4	6.14E+3	7.24E+3	1.56E+3	1.10E+3

[a] Unit = $mg \cdot L^{-1}$.

Supplementary Table 14. Water parameters of real water samples.

Water	Tap water	Lake water	Municipal Wastewater
pH	7.5	7.9	8.6
Total alkalinity ($mg{\cdot}L^{-1}$ $CaCO_3$)	15.5	63.2	120.2
UV_{254}	0.021	0.082	0.14
DOC ($mg{\cdot}L^{-1}$)	0.72	2.41	9.92
Cl^- ($mg{\cdot}L^{-1}$)	13.5	11.2	23.4
Total nitrogen (mg L^{-1})	0.35	2.08	34.56

Real environmental water in this work includes tap water, lake water, and secondary effluent from a municipal wastewater treatment plant.

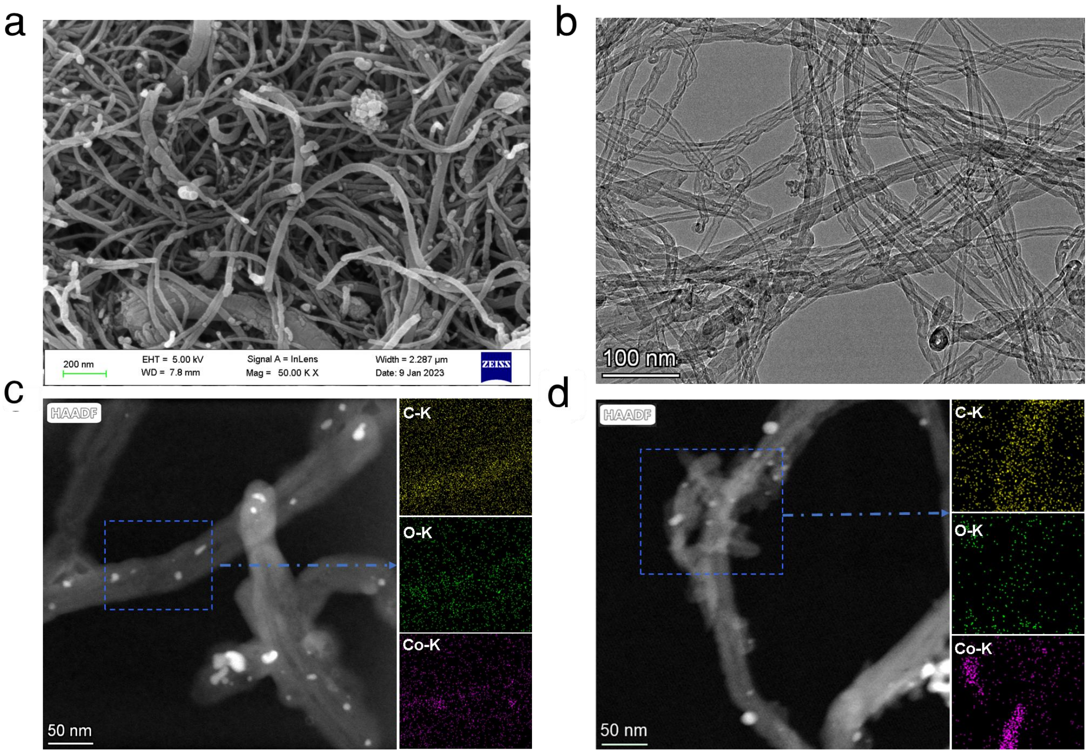

Supplementary Figure 1. (**a**) SEM images, and (**b**) TEM images of CNTs. Representative HADDF-STEM images of (**c**) Co_3O_4-*in*-CNT and (**d**) Co_3O_4-*in*-CNT, and the corresponding EDX elemental mappings of the selected area.

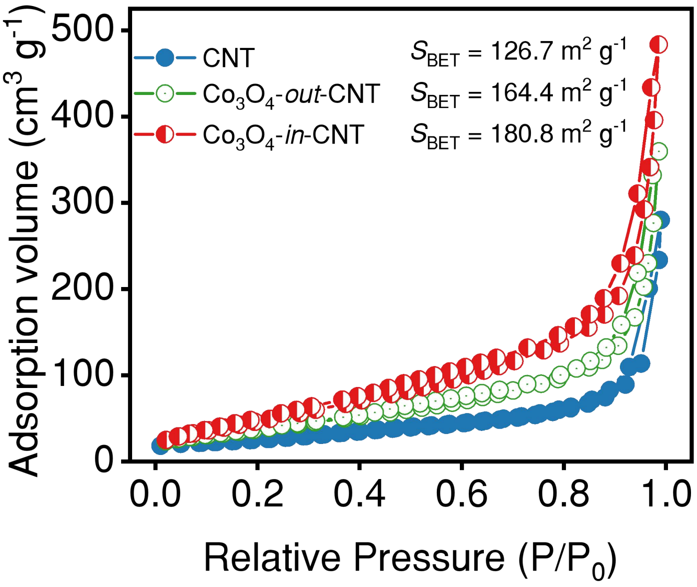

Supplementary Figure 2. N_2 adsorption-desorption isotherms.

The smooth surface of CNTs becomes rough during activation treatment (acidification and heat treatment) due to interruption or local destruction, which reduced the pore volume and diameter, and increased the surface area compared to pristine CNTs (Supplementary Table 2).

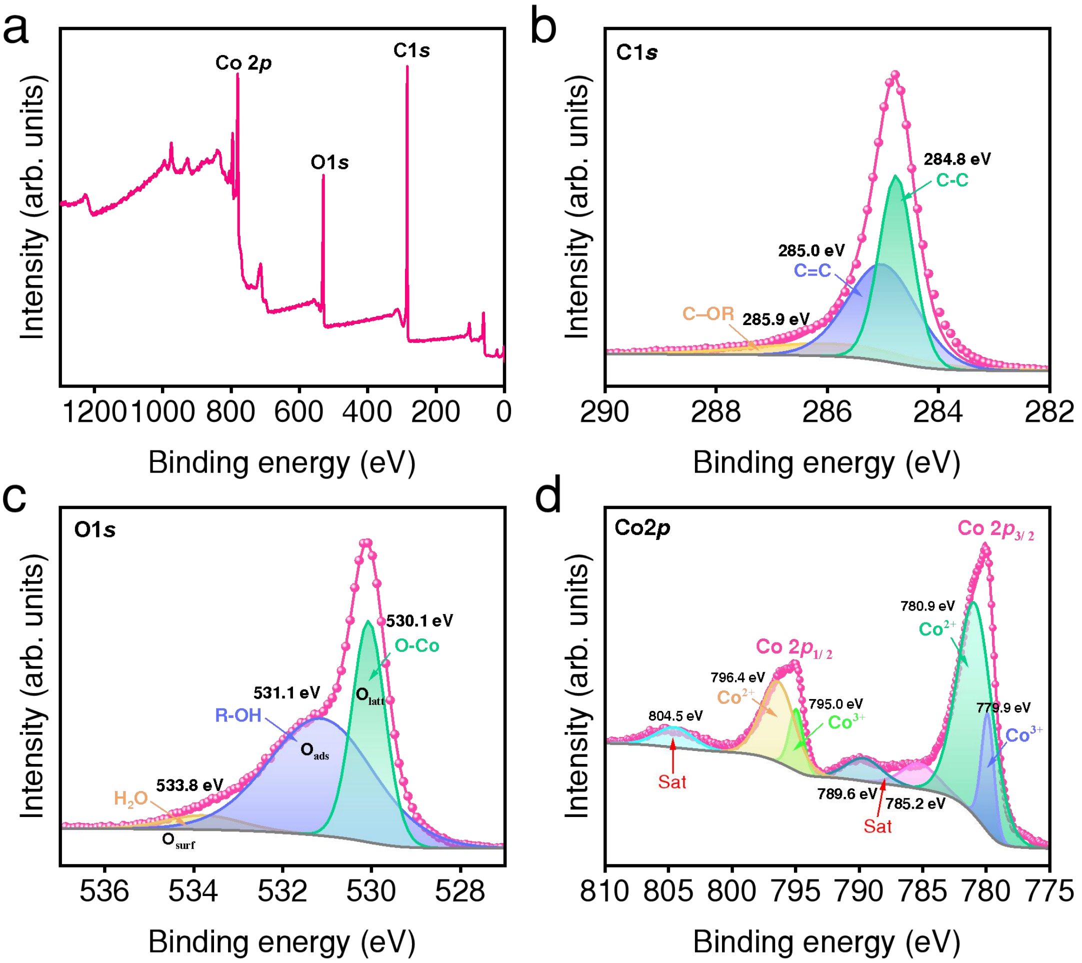

Supplementary Figure 3. XPS spectra of Co_3O_4-*in*-CNT, (**a**) the full survey scan, and high-resolution XPS spectra of (**b**) C1*s*, (**c**) O1*s* and (**d**) Co2*p*.

The high-resolution Co 2*p* XPS spectra exhibit two sharp peaks, assigning to Co 2*p*3/2 and Co 2*p*1/2, respectively. The characteristic peaks at 785.2 eV, 789.6 eV and 804.5 eV are oscillating satellite peaks, which are mainly caused by Co^{2+} oxidation states and oxygen vacancies [58]. The binding energies of 779.9/795.0 eV, and 780.9/796.4 eV could be assigned to Co^{3+} species and Co^{2+}, respectively [59]. The high resolution C1*s* can be divided into three peaks at 284.8 eV, 285.0 eV and 285.9 eV, which could be assigned to the characteristics of graphitic carbon (C-C), sp^2 carbon (C=C) and C–OR (including C–O–C and C–O–H), respectively [60]. The O1*s* spectra could be deconvoluted into three parts: lattice oxygen bonding with metal (O_{latt}, 529.9−530.5 eV), the oxygen species adsorbed on the surface (O_{ads}, 531.1-531.9 eV), and the surface-adsorbed molecular H_2O (O_{surf}, 532.7-533.8 eV). The O_{ads} peak located at 531.1 eV mainly includes O_2^{2-} or O^- in forms of hydroxyl OH^- [61].

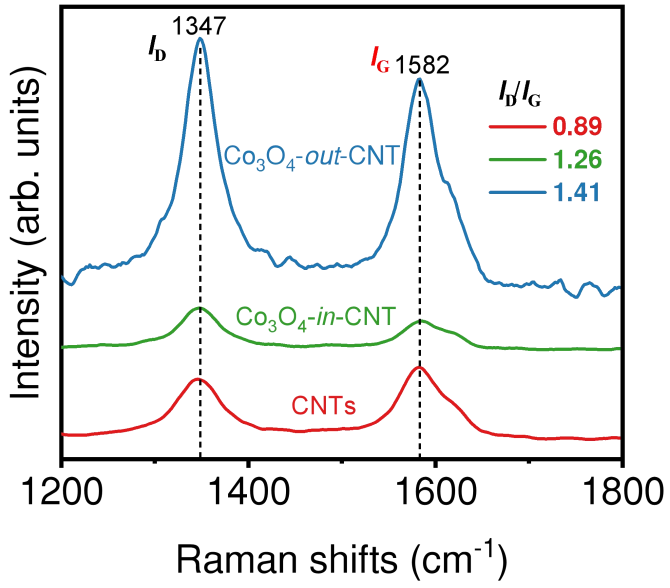

Supplementary Figure 4. The intensity of I_D/I_G of CNT (pink), Co_3O_4-*out*-CNT (yellow) and Co_3O_4-*in*-CNT (blue).

The I_D/I_G intensity ratio indicates the disorientated degree of graphene arising from the structural defects and heteroatom doping [62]. Generally, the increase of I_D/I_G represented the higher defective degree in catalysts, which could increase the catalytic activity [62].

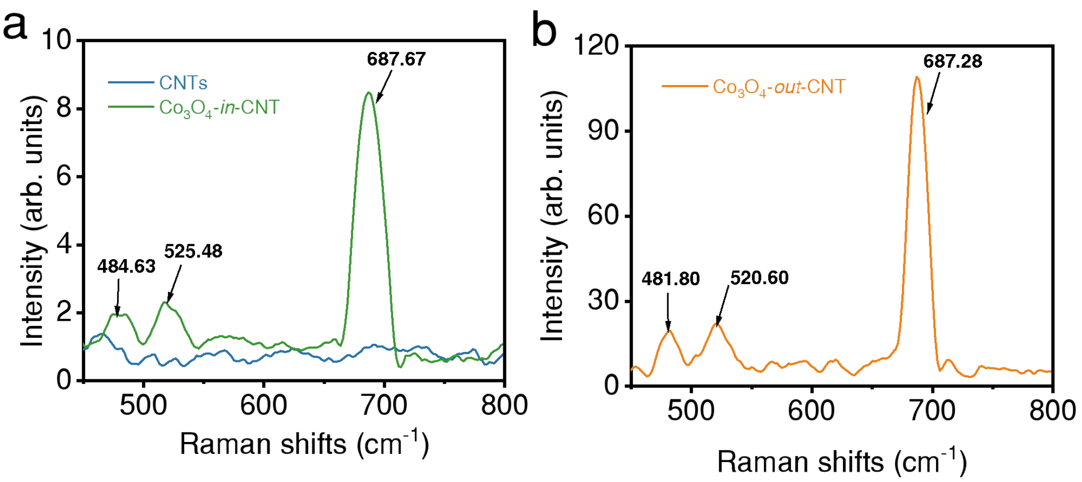

Supplementary Figure 5. Three new Raman bands appear at 400-800 cm^{-1} after deposition of Co_3O_4 NPs on the outer or inner surface of CNTs.

Specifically, the Eg mode of Co-O in the spectrum of Co_3O_4-*in*-CNT (525.5 cm^{-1}) was upshifted by 5 cm^{-1} relative to that of Co_3O_4 NPs on the outer surface of CNTs (520.6 cm^{-1}), which is consistent with the previous reported phenomenon for metal oxides confined within CNTs [63]. Hence, the upward shift of the v_{Co-O} Eg mode is attributed to the unique interaction between the Co_3O_4 NPs and the inner wall of the CNTs.

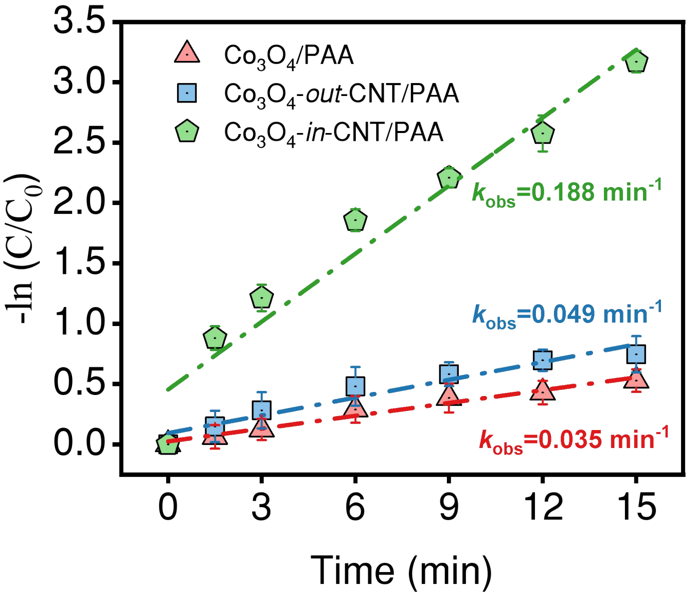

Supplementary Figure 6. Determination of apparent rate constant of BPA in the Co_3O_4/PAA, Co_3O_4-*out*-CNT/PAA and Co_3O_4-*in*-CNT/PAA systems. $[PAA]_0$ = 200 μM, $[catalysts]_0$ = 0.1 g L^{-1}, $[BPA]_0$ = 100 μM, pH = 7.0. The error bars in the figures represent the standard deviations from triplicate tests.

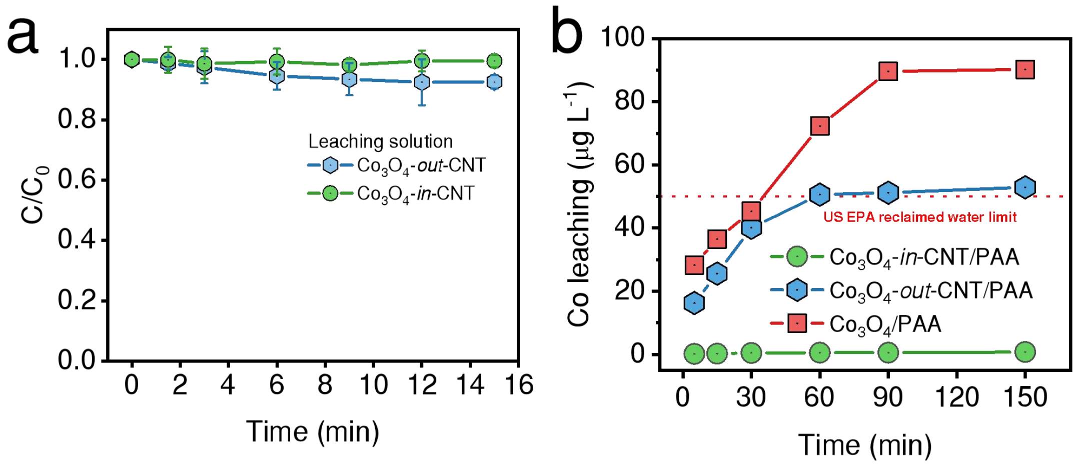

Supplementary Figure 7. (**a**) Removal of BPA with filtrate. (**b**) The leached concentration of Co ions after reaction for 150 min. $[PAA]_0$ = 200 μM, $[catalysts]_0$ = 0.1 g L^{-1}, $[BPA]_0$ = 100 μM, pH = 7.0. The error bars in the figures represent the standard deviations from triplicate tests.

The Fenton-like activity was found to be mainly derived from Co sites by comparing with the degradation kinetic of BPA in CNTs without Co active centers. Meanwhile, the leaching solution collected from of catalysts showed poor activity for the activation of PAA, indicating that the degradation of BPA was induced by heterogeneous catalytic reaction. In addition, the leached Co was remarkably reduced to 0.5 μg L^{-1} in the Co_3O_4-*in*-CNT/PAA system, which was much lower than the health reference level of Co (70 μg L^{-1}) in drinking water and the recommended limits in reclaimed water (50 μg L^{-1}) by US EPA.

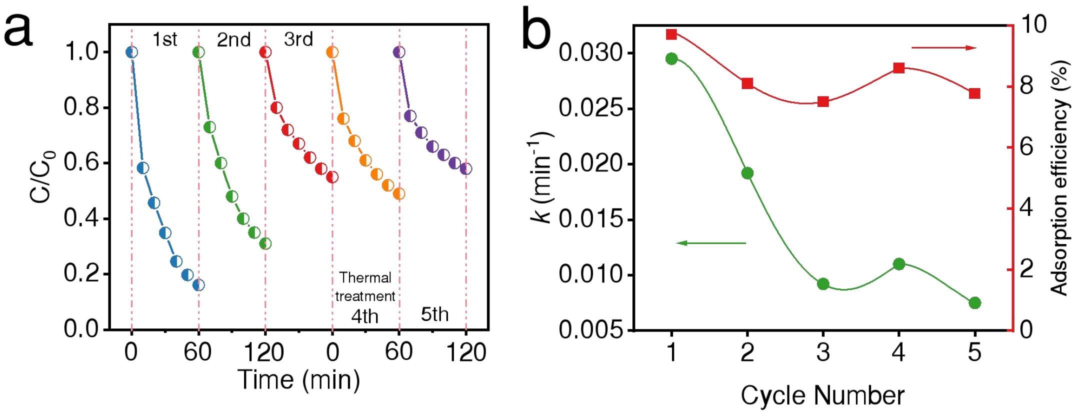

Supplementary Figure 8. (a) The evolution of catalytic activity for Co_3O_4-*out*-CNT in the cycling tests, and thermal treatment at 932.2 K for 2 h in N_2 was used to regenerate the catalytic activity after the 3rd cycle. **(b)** Relationships between the catalytic activity of Co_3O_4-*out*-CNT and the effectiveness of BPA adsorption in consecutive runs. $[PAA]_0$ = 200 μM, $[Co_3O_4$-*out*-CNT$]_0$ = 0.1 g L^{-1}, $[BPA]_0$ = 100 μM, pH = 7.0.

The progressive deactivation of the catalyst in the Co_3O_4-*out*-CNT/PAA system may also be caused by the adsorption of BPA and intermediate. After three cycles, the BPA removal was seen to steadily decline to 55%, however, the efficacy of BPA removal could be slightly improved by using thermal treatment to remove surface adsorbates. Furthermore, the effectiveness of BPA adsorption was reduced as the number of catalyst cycles increased, but the total impact of BPA adsorption on its degradation was not obvious (< 10%) (Supplementary Figure 8b). Hence the adsorption of BPA and its products is one of the factors for the catalyst deactivation, but it is not the dominant one.

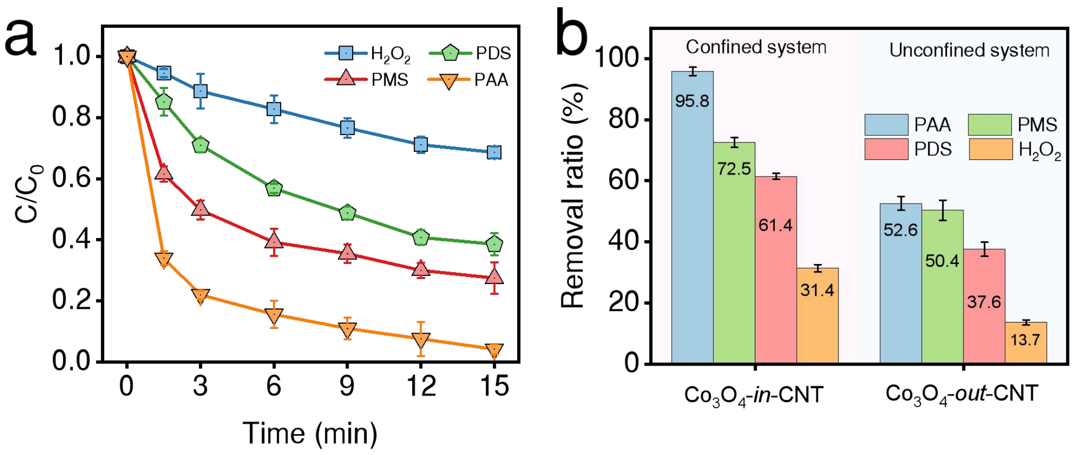

Supplementary Figure 9. (a) Effects of different oxidants with Co_3O_4-*in*-CNT on the degradation of BPA. (b) Comparison of the removal rate of BPA between Co_3O_4-*in*-CNT/peroxides and Co_3O_4-*out*-CNT/peroxides. $[PAA]_0 = [PMS]_0 = [PDS]_0 = [H_2O_2]_0 = 200$ μM, $[Co_3O_4\text{-}out\text{-}CNT]_0 = 0.1$ g L^{-1}, $[BPA]_0 = 100$ μM, $[MeOH]_0 = [TBA]_0 = 100$ mM, pH = 7.0. The error bars in the figures represent the standard deviations from triplicate tests.

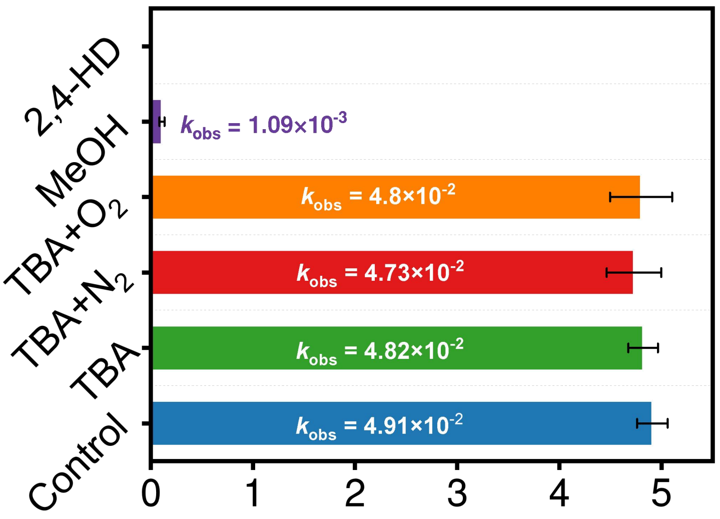

Supplementary Figure 10. Effects of different quenching agents on BPA degradation in the Co_3O_4-*out*-CNT/PAA process. $[PAA]_0$ = 200 μM, $[Co_3O_4\text{-}out\text{-}CNT]_0$ = 0.1 g L^{-1}, $[BPA]_0$ = 100 μM, $[MeOH]_0$ = $[TBA]_0$ = 100 mM, $[2,4\text{-}HD]_0$ = 5 mM, pH = 7.0. The error bars in the figures represent the standard deviations from triplicate tests.

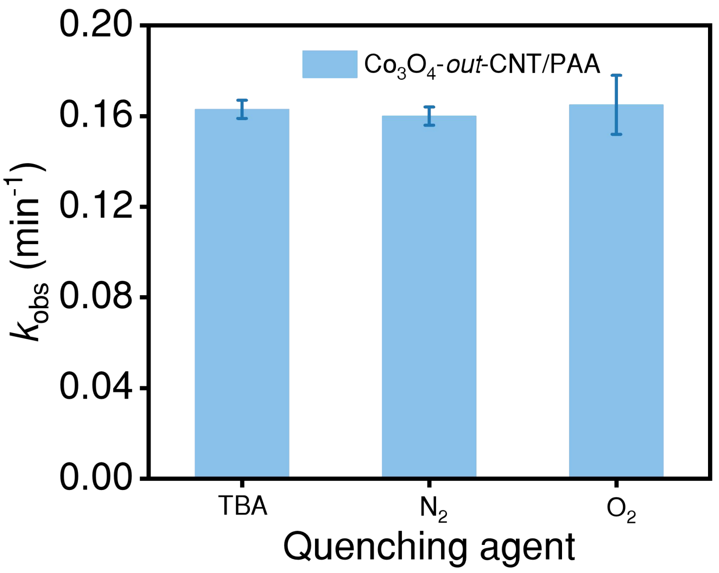

Supplementary Figure 11. Effects of DO on BPA degradation in the Co_3O_4-*out*-CNT/PAA/TBA system via purging with N_2 and O_2 for 30 min. $[PAA]_0$ = 200 μM, $[BPA]_0$ = 100 μM, $[Co_3O_4\text{-}out\text{-}CNT]_0$ = 0.1 g L^{-1}, pH = 7.0. The error bars in the figures represent the standard deviations from triplicate tests.

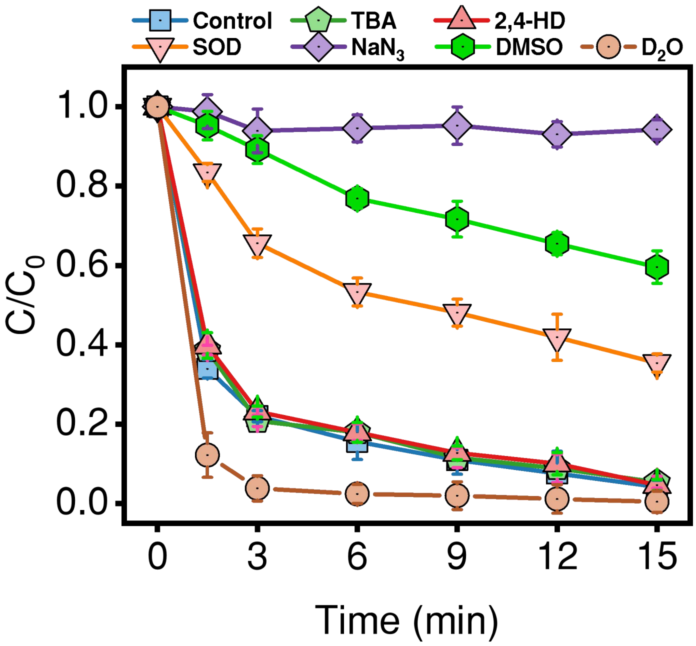

Supplementary Figure 12. Impact of scavengers on BPA degradation in the Co_3O_4-*in*-CNT/PAA system. $[PAA]_0$ = 200 μM, $[Co_3O_4\text{-}in\text{-}CNT]_0$ = 0.1 g L^{-1}, $[BPA]_0$ = 100 μM, $[TBA]_0$ = 100 mM, $[NaN_3]_0$ = 10 mM, $[DMSO]_0$ = $[2,4\text{-}HD]_0$ = 5 mM, pH = 7.0. The error bars in the figures represent the standard deviations from triplicate tests.

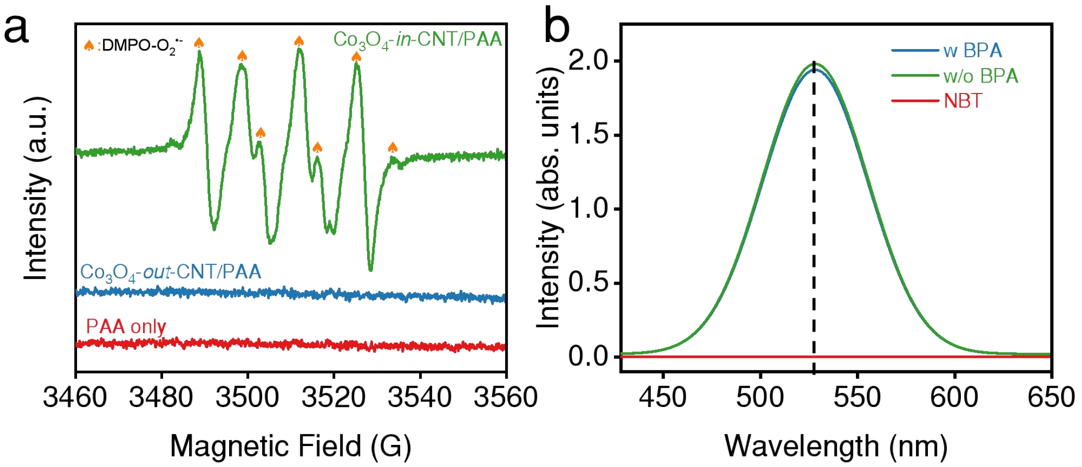

Supplementary Figure 13. (**a**) DMPO was used as the spin-trapping agent in the EPR spectra of the PAA only, Co_3O_4-*out*-CNT/PAA and Co_3O_4-*in*-CNT/PAA systems, which were recorded at a reaction time of 5 min (the yellow heart represents the DMPO-$O_2^{\bullet -}$ adducts). (**b**) UV-vis adsorption spectra of NBT in the Co_3O_4-*in*-CNT/PAA system. $[PAA]_0$ = 200 μM, $[Co_3O_4$-*out*-$CNT]_0$ = $[Co_3O_4$-*in*-$CNT]_0$ = 0.1 g L^{-1}, $[BPA]_0$ = 100 μM, $[DMPO]_0$ = 100 mM, pH = 7.0.

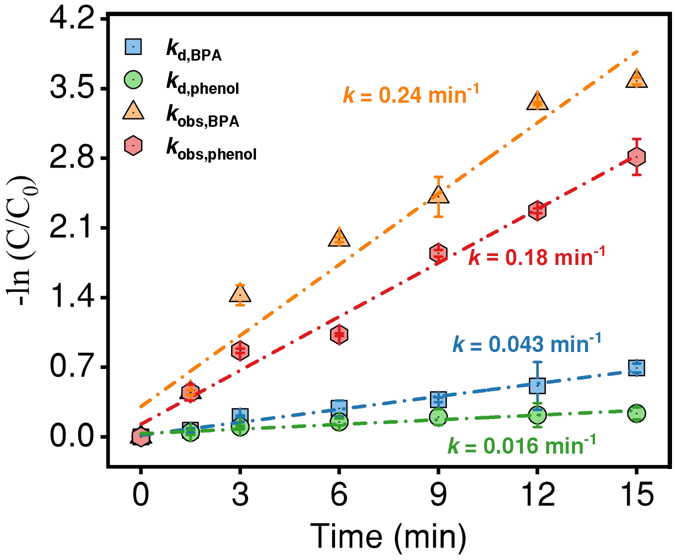

Supplementary Figure 14. Determination of second-order rate constants of BPA with $O_2^{\bullet-}$ using phenol as a competitor under UV irradiation. Reaction conditions: $[BPA]_0$ = $[phenol]_0$ = 50 μM. Note: the solutions containing $O_2^{\bullet-}$ were prepared from the photolysis of oxygen-saturated solutions with 41.0 mM acetone, 12.0 M 2-propanol, 15.0 μM diethylenetriaminepentaacetic acid (DTPA) and 1.0 mM borate buffer. The error bars in the figures represent the standard deviations from triplicate tests.

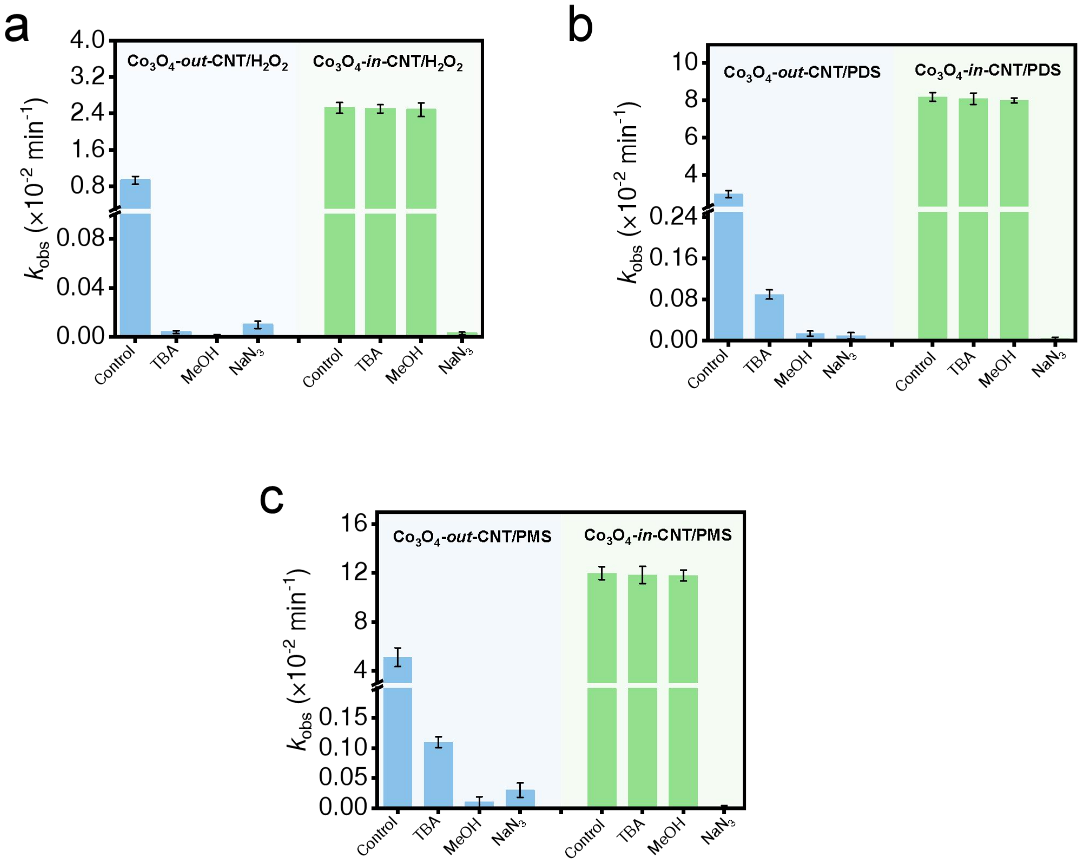

Supplementary Figure 15. Effects of different quenching agents on BPA degradation in the (**a**) H_2O_2, (**b**) PDS, and (**c**) PMS activation processes. $[H_2O_2]_0$ = $[PDS]_0$ = $[PMS]_0$ = 200 μM, $[Co_3O_4$-*out*-CNT$]_0$ = $[Co_3O_4$-*in*-CNT$]_0$ = 0.1 g L^{-1}, $[BPA]_0$ = 100 μM, $[MeOH]_0$ = $[TBA]_0$ = 100 mM, $[NaN_3]_0$ = 10 mM, pH = 7.0. In the figure, the shading base colors light blue and light yellow represent the Co_3O_4-*out*-CNT/peroxides and Co_3O_4-*in*-CNT/peroxides systems, respectively. The error bars in the figures represent the standard deviations from triplicate tests.

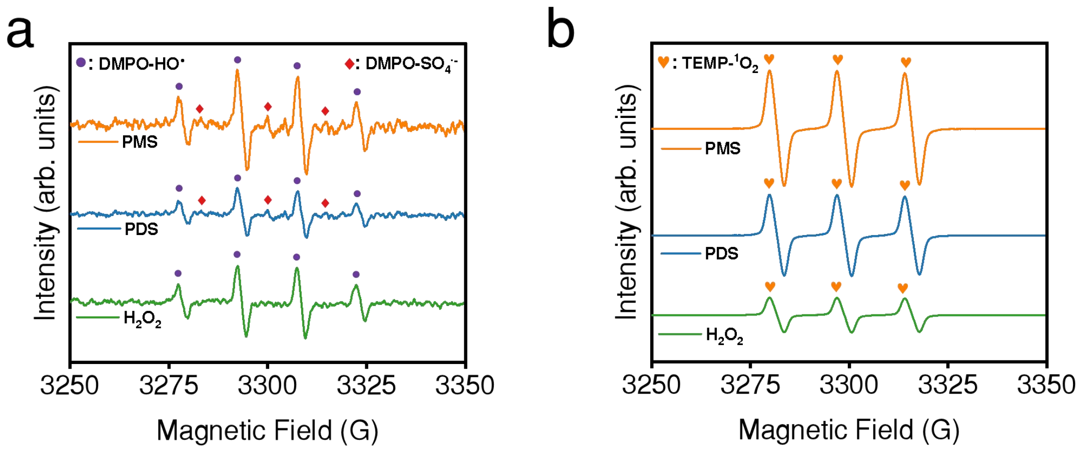

Supplementary Figure 16. EPR spectra of (**a**) DMPO- HO$^{•}$ or DMPO-$SO_4^{•-}$ (purple dots and red diamonds indicate the DMPO- HO$^{•}$ and DMPO-$SO_4^{•-}$ adducts, respectively) and (**b**) TEMP–$^1O_2$ adducts (yellow hearts indicate the TEMP-$^1O_2$ adducts) were detected in the Co_3O_4-*out*-CNT/peroxides and Co_3O_4-*in*-CNT/peroxides systems, respectively. $[H_2O_2]_0$ = $[PDS]_0$ = $[PMS]_0$ = 200 μM, $[Co_3O_4\text{-}out\text{-}CNT]_0$ = $[Co_3O_4\text{-}in\text{-}CNT]_0$ = 0.1 g L^{-1}, $[DMPO]_0$ = $[TEMP]_0$ = 100 mM, pH = 7.0.

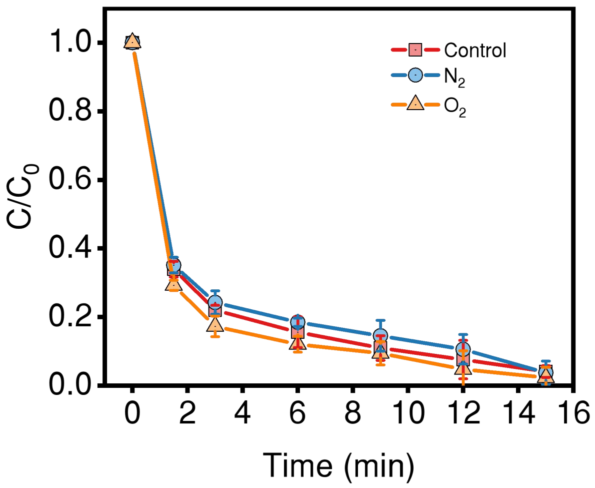

Supplementary Figure 17. Effects of DO on BPA degradation in the Co_3O_4-*in*-CNT/PAA system via purging with N_2 and O_2 for 30 min. $[PAA]_0$ = 200 μM, $[BPA]_0$ = 100 μM, $[Co_3O_4\text{-}in\text{-}CNT]_0$ = 0.1 g L^{-1}, pH = 7.0. The error bars in the figures represent the standard deviations from triplicate tests.

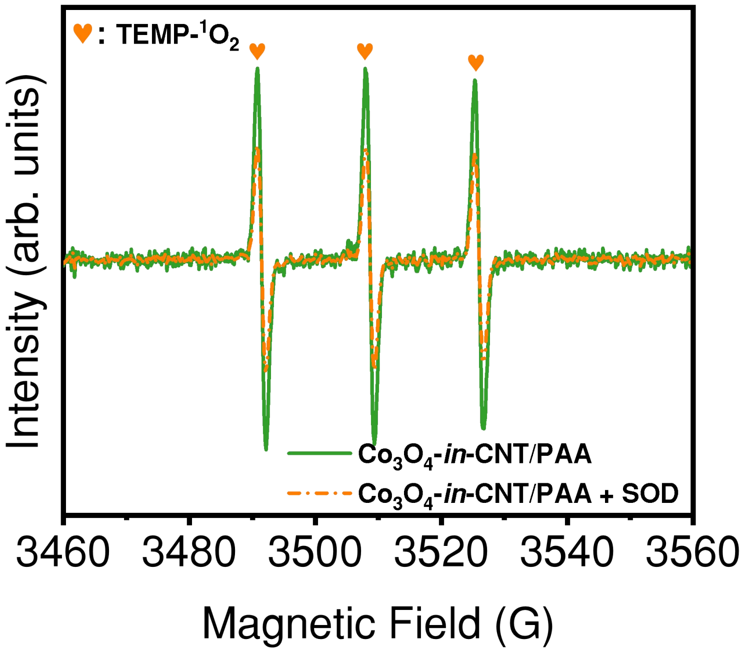

Supplementary Figure 18. EPR spectra of TEMP-$^1O_2$ adducts (yellow hearts indicate the TEMP-$^1O_2$ adducts) in the Co_3O_4-*in*-CNT/PAA system with or without SOD. $[PAA]_0$ = 200 μM, $[TEMP]_0$ = 100 mM, $[Co_3O_4\text{-}in\text{-}CNT]_0$ = 0.1 g L^{-1}, pH = 7.0.

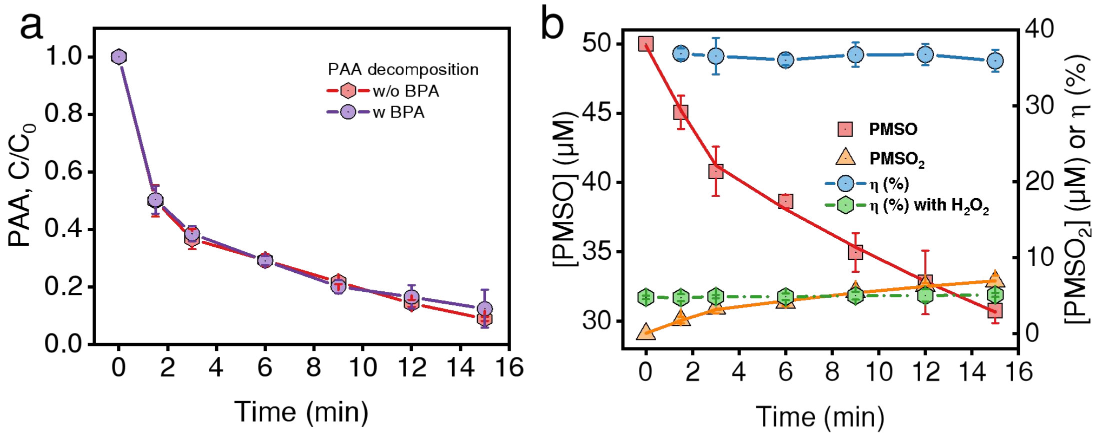

Supplementary Figure 19. (**a**) Decomposition of PAA with and without BPA in the Co_3O_4-*in*-CNT/PAA system. (**b**) PMSO degradation, $PMSO_2$ production, and the molar yield of $PMSO_2$ (η) in the Co_3O_4-*in*-CNT/PAA system. $[PAA]_0$ = 200 μM, $[BPA]_0$ = 100 μM, $[Co_3O_4\text{-}in\text{-}CNT]_0$ = 0.1 g L^{-1}, $[PMSO]_0$ = 50 μM, $[H_2O_2]_0$ = 1.0 mM, pH = 7.0. The error bars in the figures represent the standard deviations from triplicate tests.

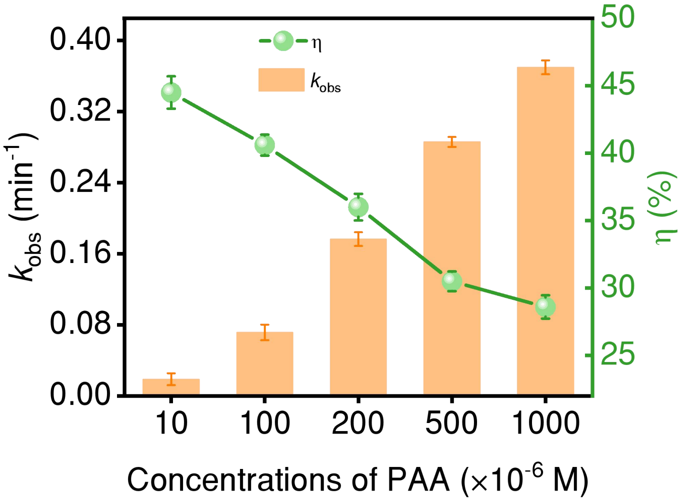

Supplementary Figure 20. Apparent degradation rate of BPA (k_{obs}) and the molar yield of $PMSO_2$ (η) with various PAA concentrations in the Co_3O_4-*in*-CNT/PAA system. $[PAA]_0$ = 100-1000 μM, $[PMSO]_0$ = 50 μM, $[Co_3O_4\text{-}in\text{-}CNT]_0$ = 0.1 g L^{-1}, pH = 7.0. The error bars in the figures represent the standard deviations from triplicate tests.

Noting the coexistence of H_2O_2 in the PAA solution, the impact of H_2O_2 on the conversion of Co(IV) was further investigated. The addition of extra H_2O_2 (1.0 mM) strengthened the generation of $O_2^{\bullet-}$ and strikingly inhibited PMSO degradation (with η-($PMSO_2$) dropping to around 5%) (Supplementary Fig. 19b), implying the competitive consumption of Co(IV) by H_2O_2. In addition, Co(IV) was reported to promote PAA decomposition, generating $^1O_2$ in the Co(II)/PAA process [64, 65]. Meanwhile, Co(IV) most likely acted as the other pivotal intermediate in the production pathway of $^1O_2$ rather than direct oxidant for BPA. Indeed, as the increase of PAA (10-100 μM), the η-($PMSO_2$) declined but the degradation of BPA was distinctly accelerated (Supplementary Fig. 20), indicating that the reaction between Co(IV) and PAA produce $^1O_2$. Overall, $^1O_2$ was originated from the transformation of crucial intermediates of $O_2^{\bullet-}$ and Co(IV) in the Co_3O_4-*in*-CNT/PAA system.

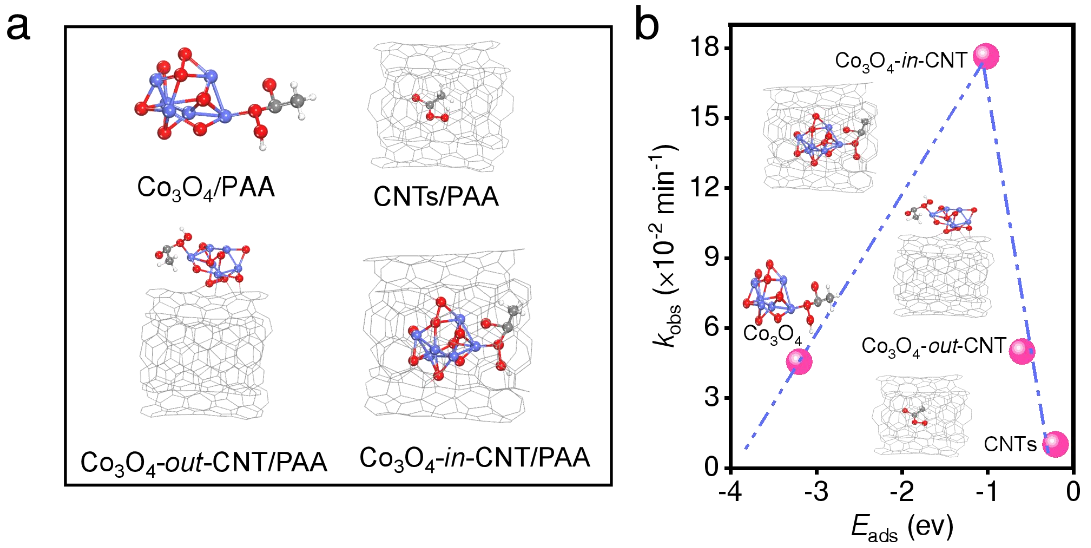

Supplementary Figure 21. Volcano plots of the reaction rate (k_{obs}) plotted as a function of the adsorption energy (E_{ads}) of PAA on CNTs, Co_3O_4-*out*-CNT**,** Co_3O_4-*in*-CNT and Co_3O_4.

Adsorption energies (E_{ads}) were calculated to shed light on the theoretical insights for PAA activation. The interaction energies of the adsorbed PAA on CNTs was -4.85 kcal mol^{-1}. Nevertheless, the corresponding interaction energies were reduced to -13.72 and -23.51 kcal mol^{-1} in the Co_3O_4-*out*-CNT and Co_3O_4-*in*-CNT, indicating that the confinement of Co_3O_4 NPs inside CNTs strongly enhanced the adsorption capacity of PAA. In addition, the plot of reaction rate (k_{obs}) with E_{ads} follows a volcano relation. that is, the binding of PAA on CNTs and Co_3O_4-*out*-CNT is too weak to effectively activate PAA for BPA degradation. Whereas, the low reactivity of PAA on Co_3O_4 NPs could be ascribed to the strong binding of PAA, which results in the active site poisoning [46].

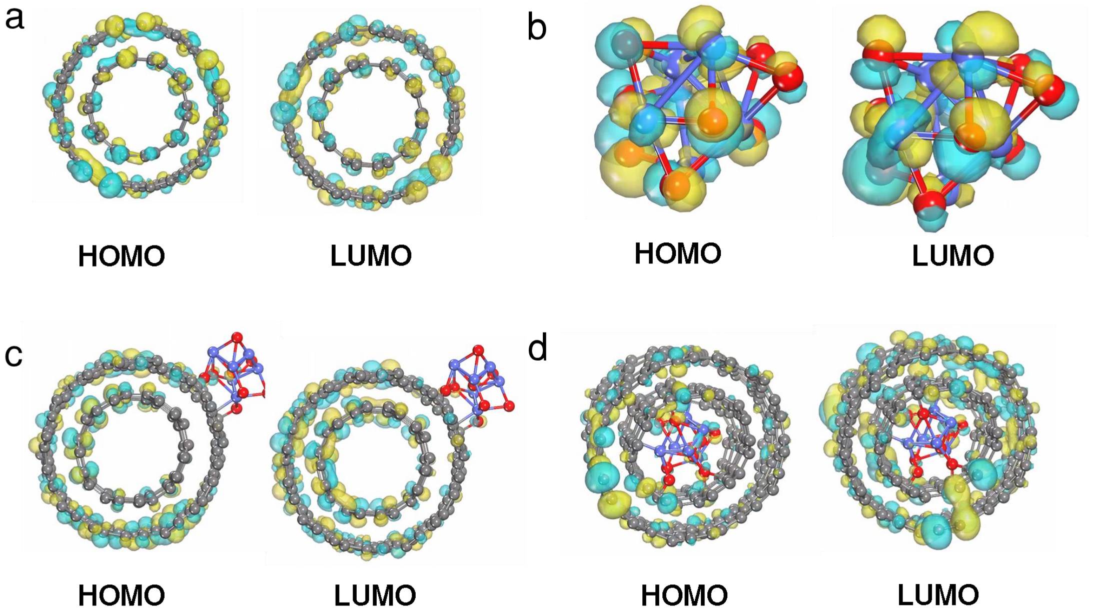

Supplementary Figure 22. HOMO and LUMO of (**a**) pristine CNTs, (**b**) Co_3O_4, (**c**) Co_3O_4-*out*-CNT, (**d**) Co_3O_4-*in*-CNT.

The HOMO electrons located in Co_3O_4-*in*-CNT (-5.27 eV) readily migrated to the LUMO of PAA (-2.72 eV), launching an electrophilic attack on the O-O bond of PAA (Table S6)[66]. The HOMO of BPA (-5.94 eV) was more negative than the LUMO of $^1O_2$ (-4.59 eV), suggesting that the electrons of BPA could migrate to the LUMO of $^1O_2$ and lead to subsequent degradation of BPA.

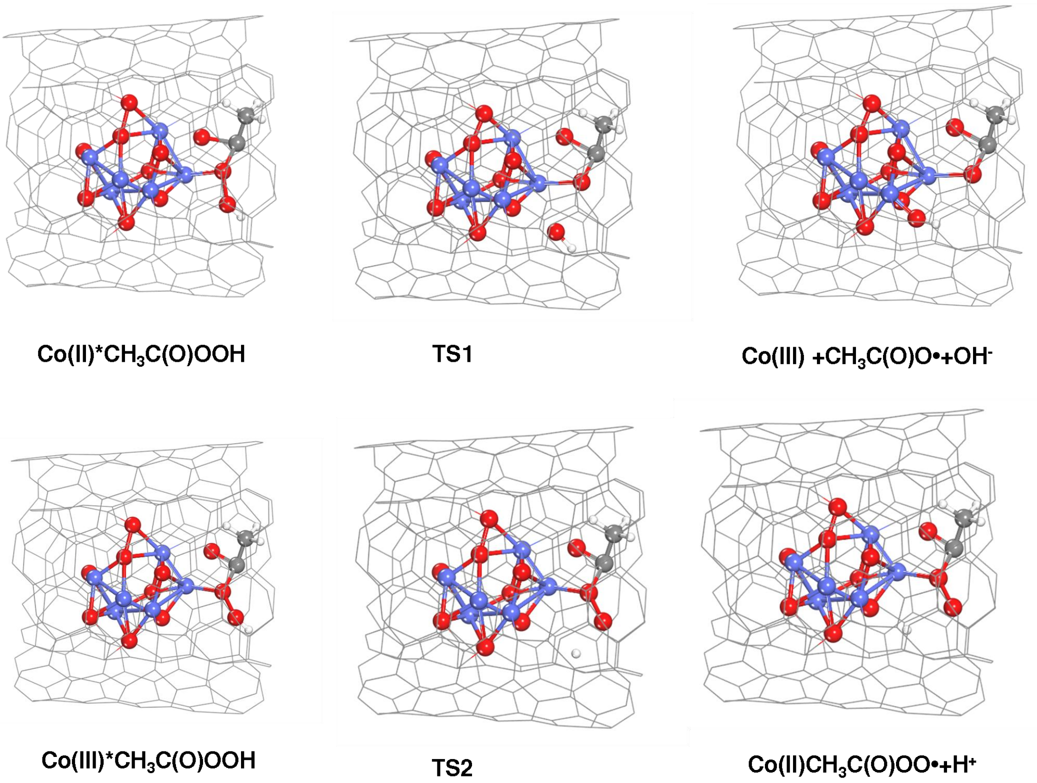

Supplementary Figure 23. Model sketches of the corresponding free energies of intermediates (INT), the energy barriers of transition states (TSs) and the final product of Co_3O_4-*out*-CNT/PAA reaction.

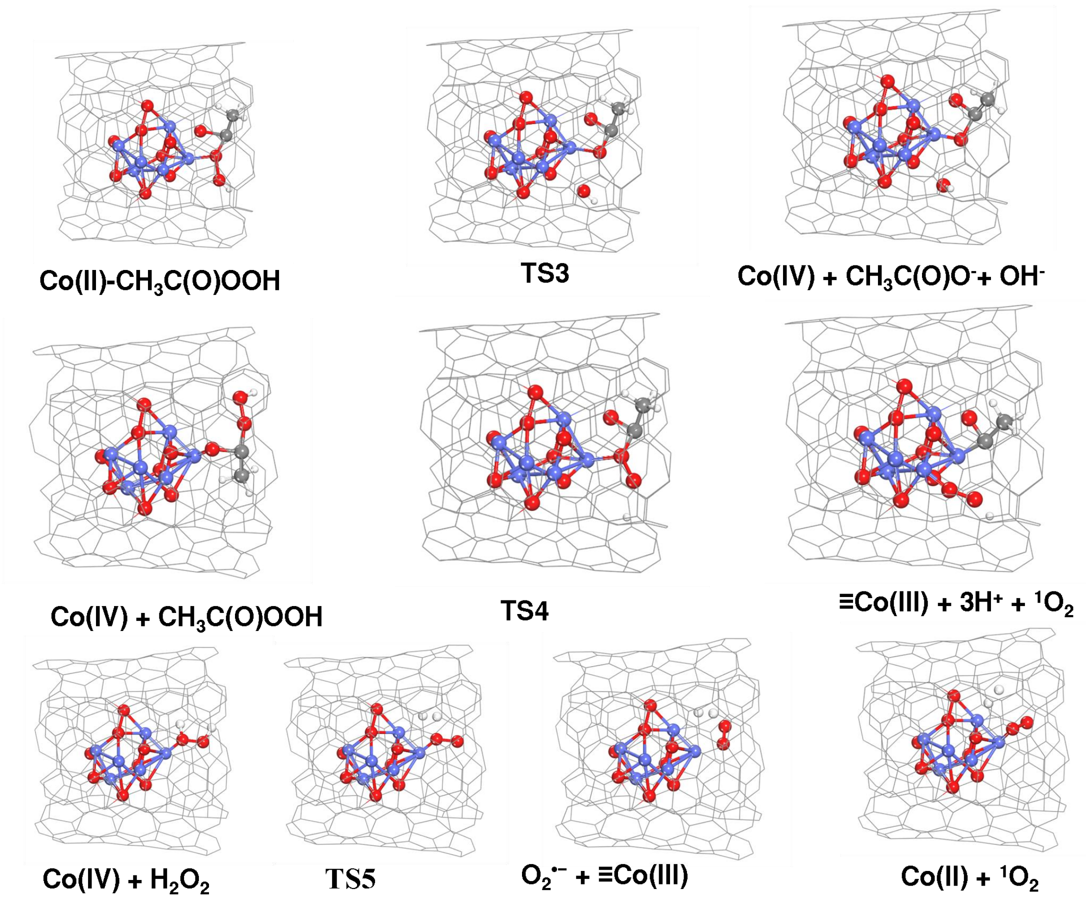

Supplementary Figure 24. Model sketches of the corresponding free energies of intermediates (INT), the energy barriers of transition states (TSs) and the final product of Co_3O_4-*in*-CNT/PAA reaction.

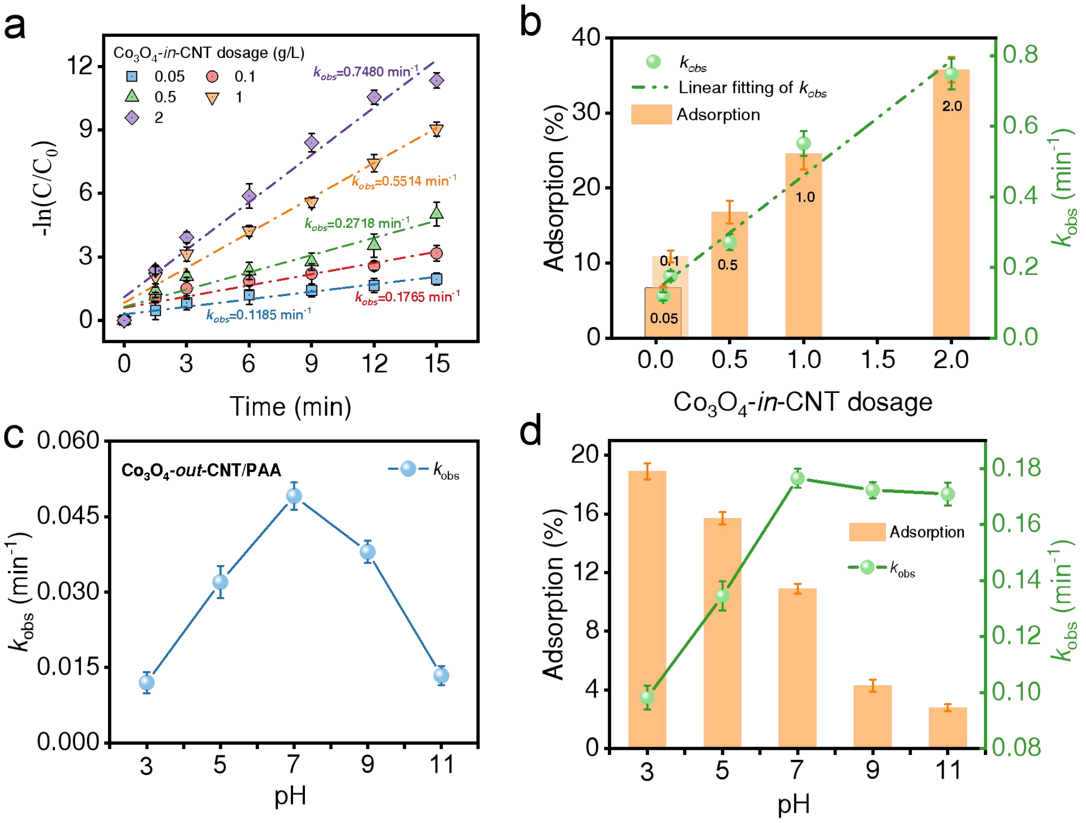

Supplementary Figure 25. (**a**) The effects of Co_3O_4-*in*-CNT dosages on BPA degradation in the Co_3O_4-*in*-CNT/PAA process; (**b**) the relationship between adsorption rates and k_{obs} of BPA under different Co_3O_4-*in*-CNT dosages; (**c**) corresponding degradation rates of BPA under different pH conditions in the Co_3O_4-*out*-CNT/PAA process; (**d**) the relationship between adsorption rates and k_{obs} of BPA under different solution in the Co_3O_4-*in*-CNT/PAA process. $[PAA]_0$ = 200 μM, $[BPA]_0$ = 100 μM, $[Co_3O_4\text{-}in\text{-}CNT]_0$ = 0.05-2 g L^{-1} for (a) and (b), $[Co_3O_4\text{-}in\text{-}CNT]_0$ = 0.1 g L^{-1} for (c) and (d), pH = 7.0 for (a) and (b), pH = 3.0-11.0 for (c) and (d). The error bars in the figures represent the standard deviations from triplicate tests.

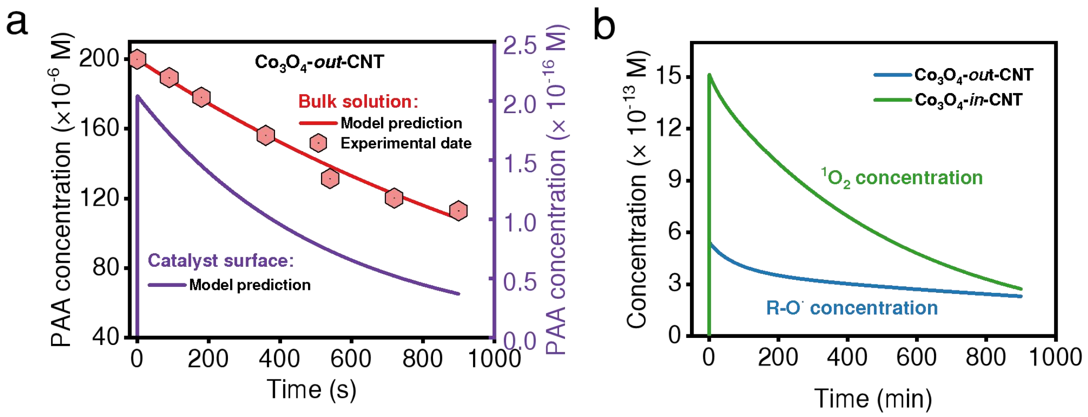

Supplementary Figure 26. (**a**) Variation of PAA concentrations on catalyst surface and in bulk solution in the Co_3O_4-*out*-CNT/PAA process. (**b**) Concentration changes of ROS in the Co_3O_4-*out*-CNT/PAA and Co_3O_4-*in*-CNT/PAA systems, respectively. $[PAA]_0$ = 200 μM, $[BPA]_0$ = 100 μM, $[Co_3O_4\text{-}in\text{-}CNT]_0$ = $[Co_3O_4\text{-}out\text{-}CNT]_0$ = 0.1 g L^{-1}, pH = 7.0.

a

b

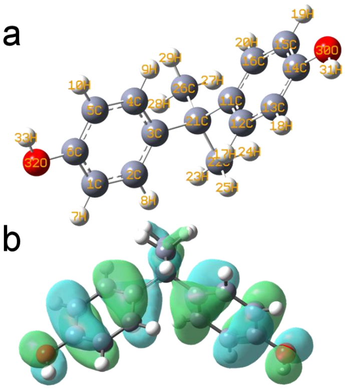

c

Atom	q (N)	q (N-1)	q (N+1)	f-	Atom	q (N)	q (N-1)	q (N+1)	f-
C1	-0.0623	-0.0090	-0.1298	0.0534	C14	0.0720	0.1259	0.0417	0.0539
C2	-0.0446	-0.0144	-0.1037	0.0302	C15	-0.0623	-0.0090	-0.1297	0.0534
C3	-0.0081	0.0399	-0.0205	0.0479	C16	-0.0446	-0.0143	-0.1037	0.0302
C4	-0.0409	-0.0099	-0.0924	0.0310	C21	0.0282	0.0325	0.0286	0.0043
C5	-0.0716	-0.0370	-0.1352	0.0346	C22	-0.0860	-0.0741	-0.0942	0.0120
C6	0.0720	0.1259	0.0417	0.0539	C26	-0.0860	-0.0741	-0.0942	0.0120
C11	-0.0081	0.0399	-0.0205	0.0480	O30	-0.1977	-0.1256	-0.2274	0.0721
C12	-0.0409	-0.0098	-0.0923	0.0311	O32	-0.1977	-0.1256	-0.2274	0.0720
C13	-0.0716	-0.0370	-0.1352	0.0346					

Supplementary Figure 27. (**a**) Optimized geometry of BPA molecule at the B3LYP/6-31G(d, p) level. (**b**) HOMO molecular orbitals in optimized geometry of BPA. (**c**) Hirshfeld charge distribution and condensed Fukui functions of the optimized BPA molecule (N+1 and N-1).

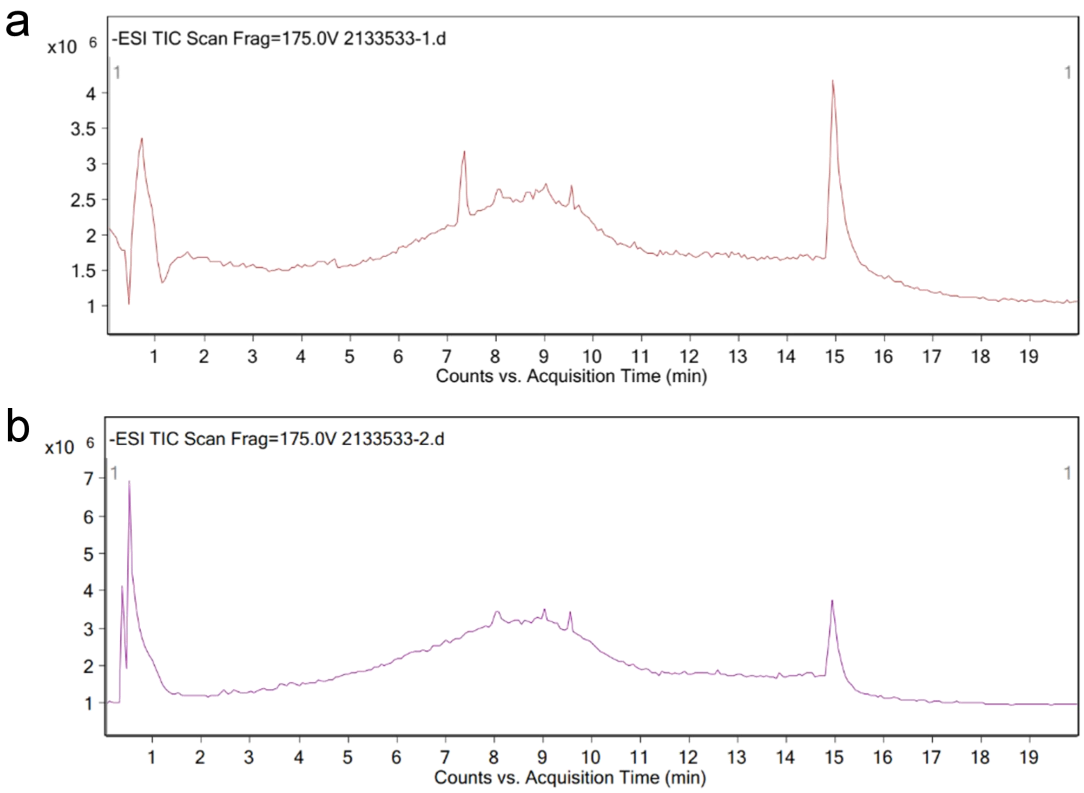

Supplementary Figure 28. Total ion chromatograms (TIC) of BPA under ESI negative mode with the reaction 0 and 15 min.

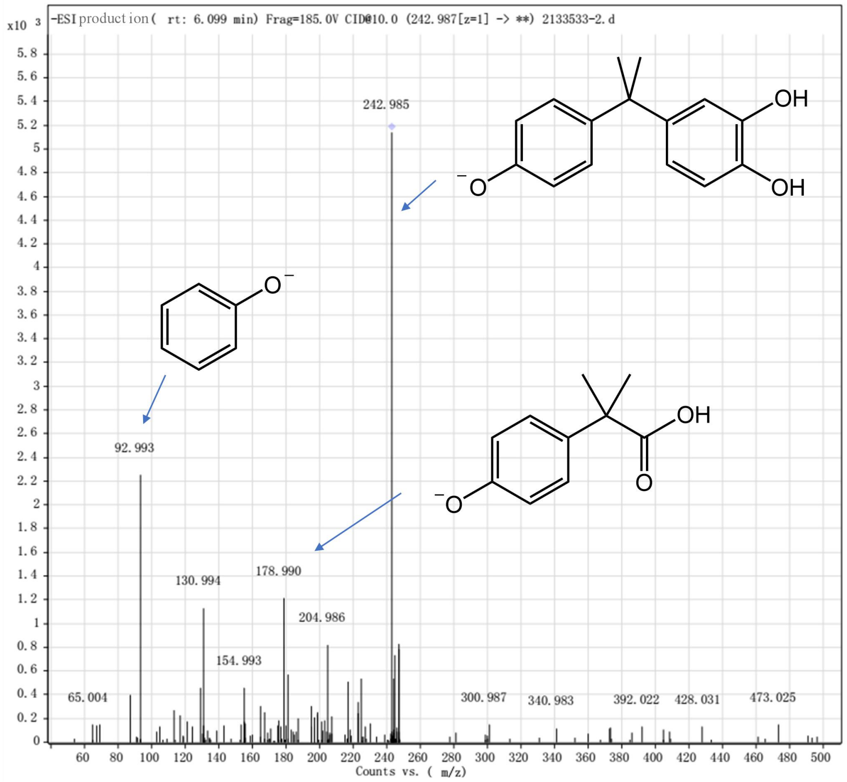

Supplementary Figure 29. The Mass spectrum of TP244 by HPLC-MS/MS (ESI-) and proposed fragmentation pathway.

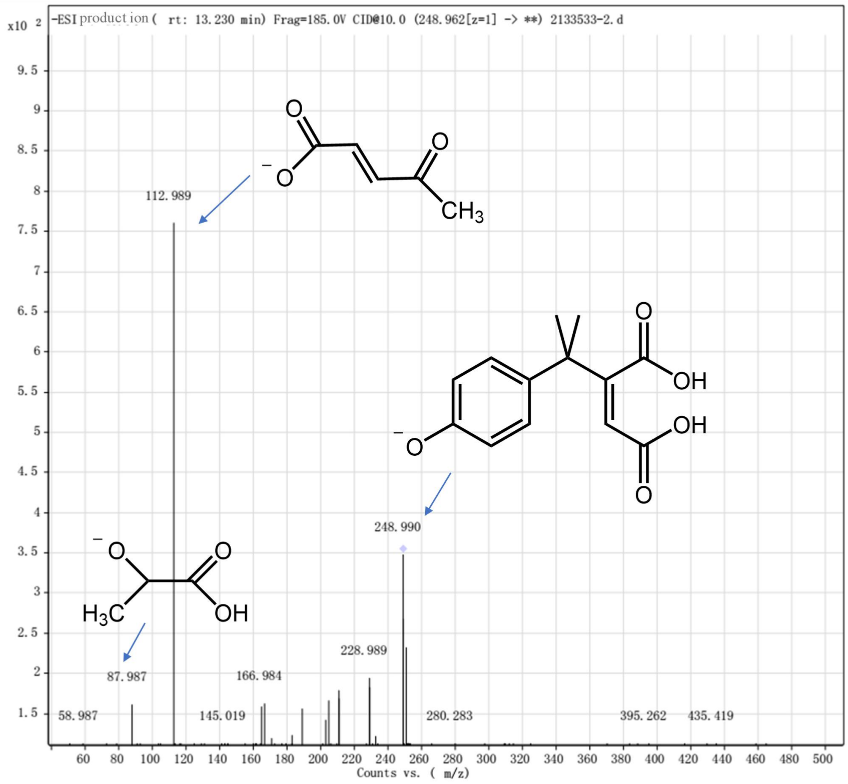

Supplementary Figure 30. The Mass spectrum of TP250 by HPLC-MS/MS (ESI-) and proposed fragmentation pathway.

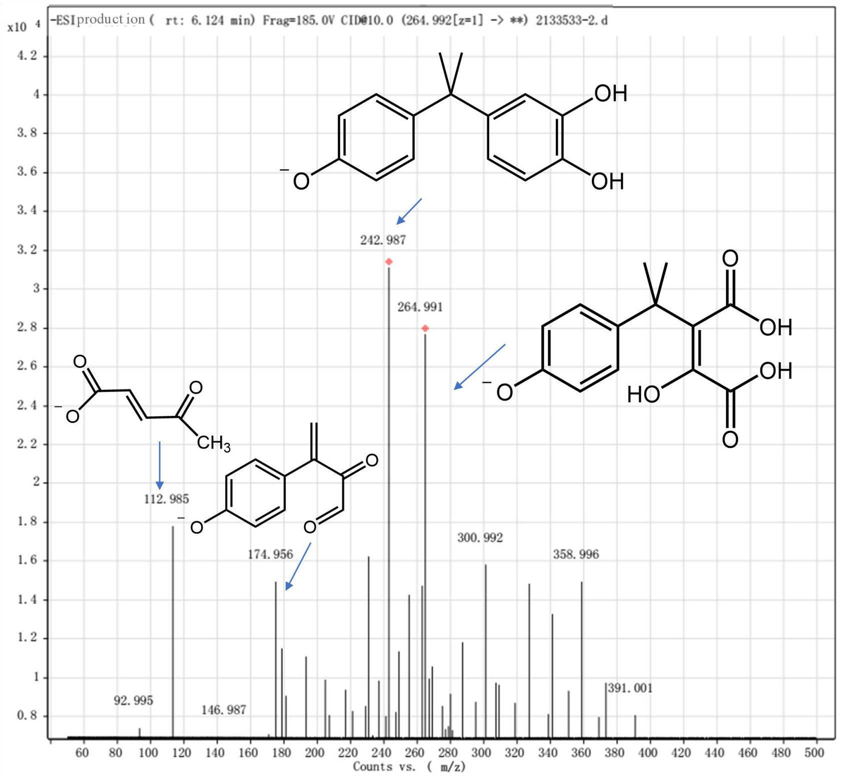

Supplementary Figure 31. The Mass spectrum of TP266 by HPLC-MS/MS (ESI-) and proposed fragmentation pathway.

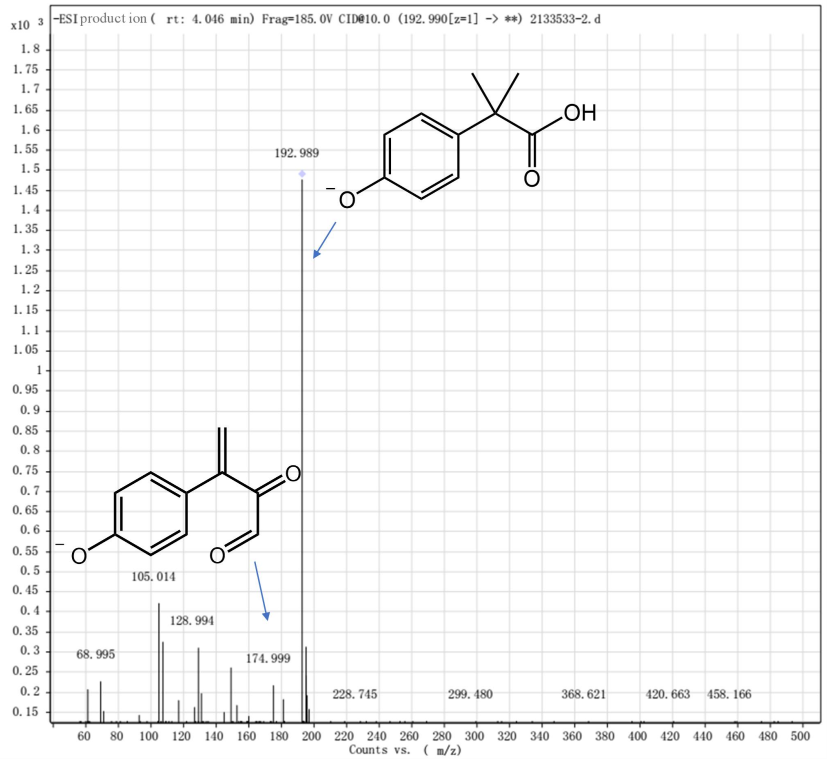

Supplementary Figure32. The Mass spectrum of TP194 by HPLC-MS/MS (ESI-) and proposed fragmentation pathway.

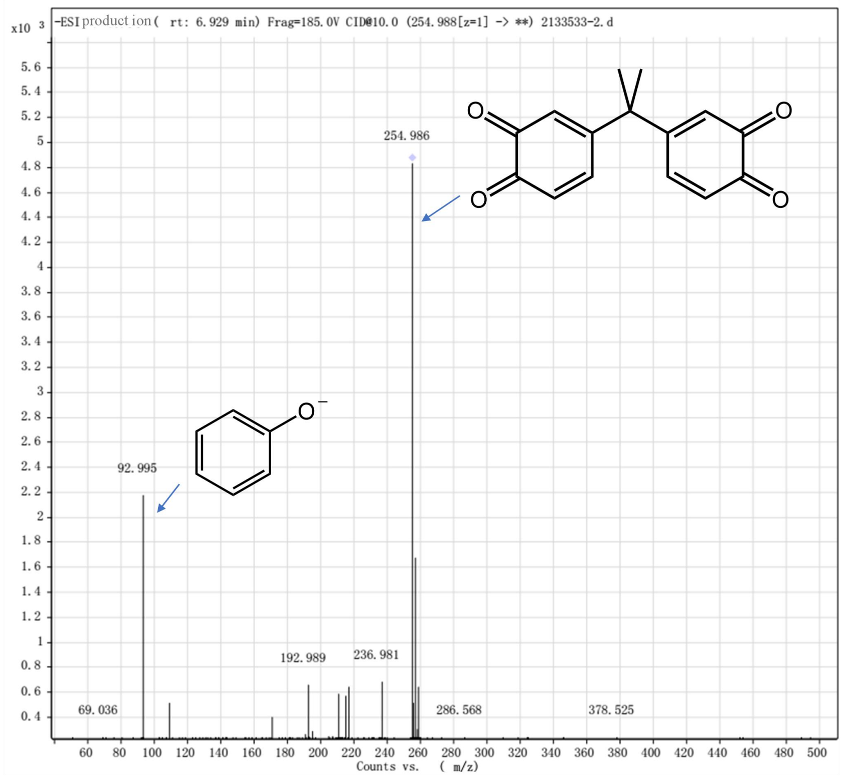

Supplementary Figure 33. The Mass spectrum of TP256 by HPLC-MS/MS (ESI-) and proposed fragmentation pathway.

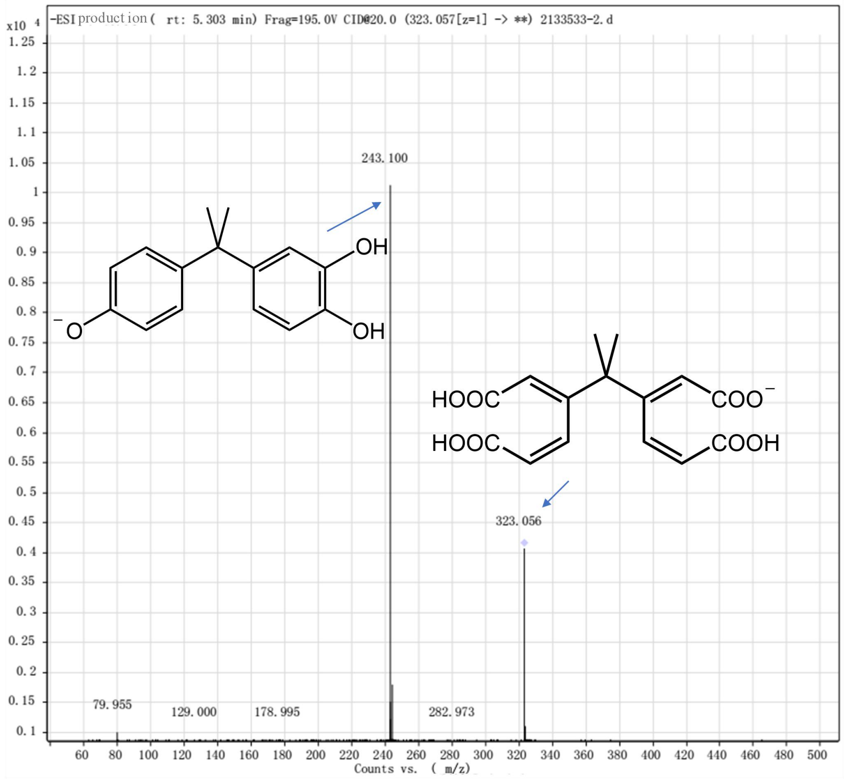

Supplementary Figure 34. The Mass spectrum of TP324 by HPLC-MS/MS (ESI-) and proposed fragmentation pathway.

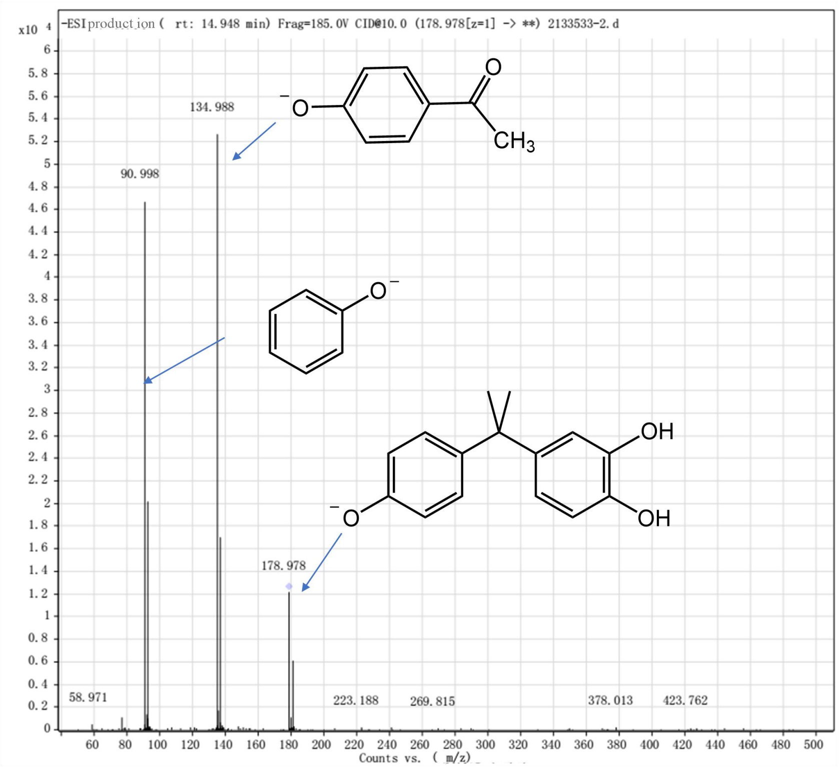

Supplementary Figure 35. The Mass spectrum of TP180 by HPLC-MS/MS (ESI-) and proposed fragmentation pathway.

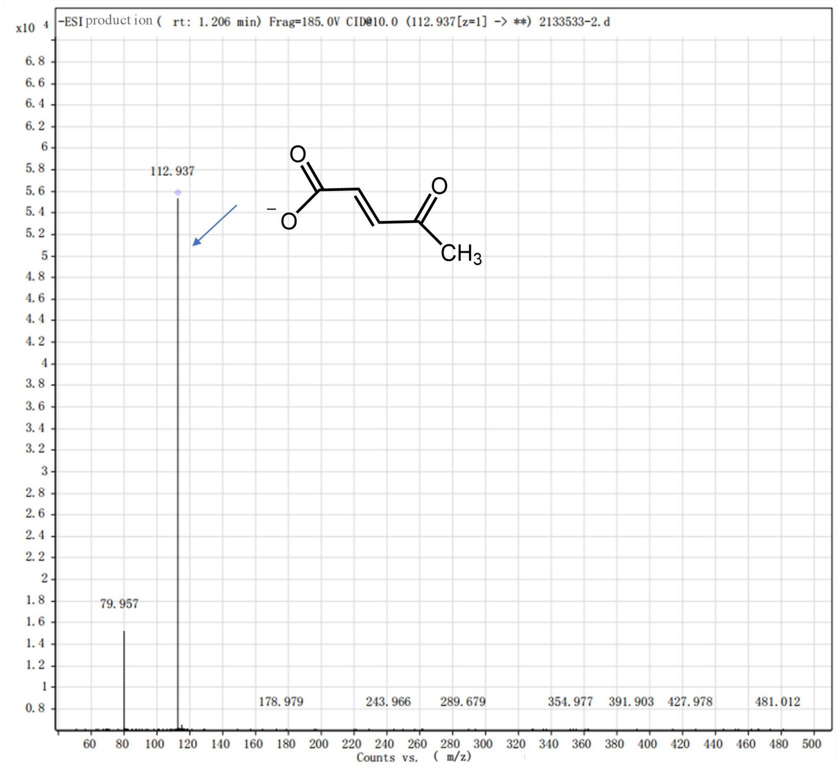

Supplementary Figure 36. The Mass spectrum of TP114 by HPLC-MS/MS (ESI-) and proposed fragmentation pathway.

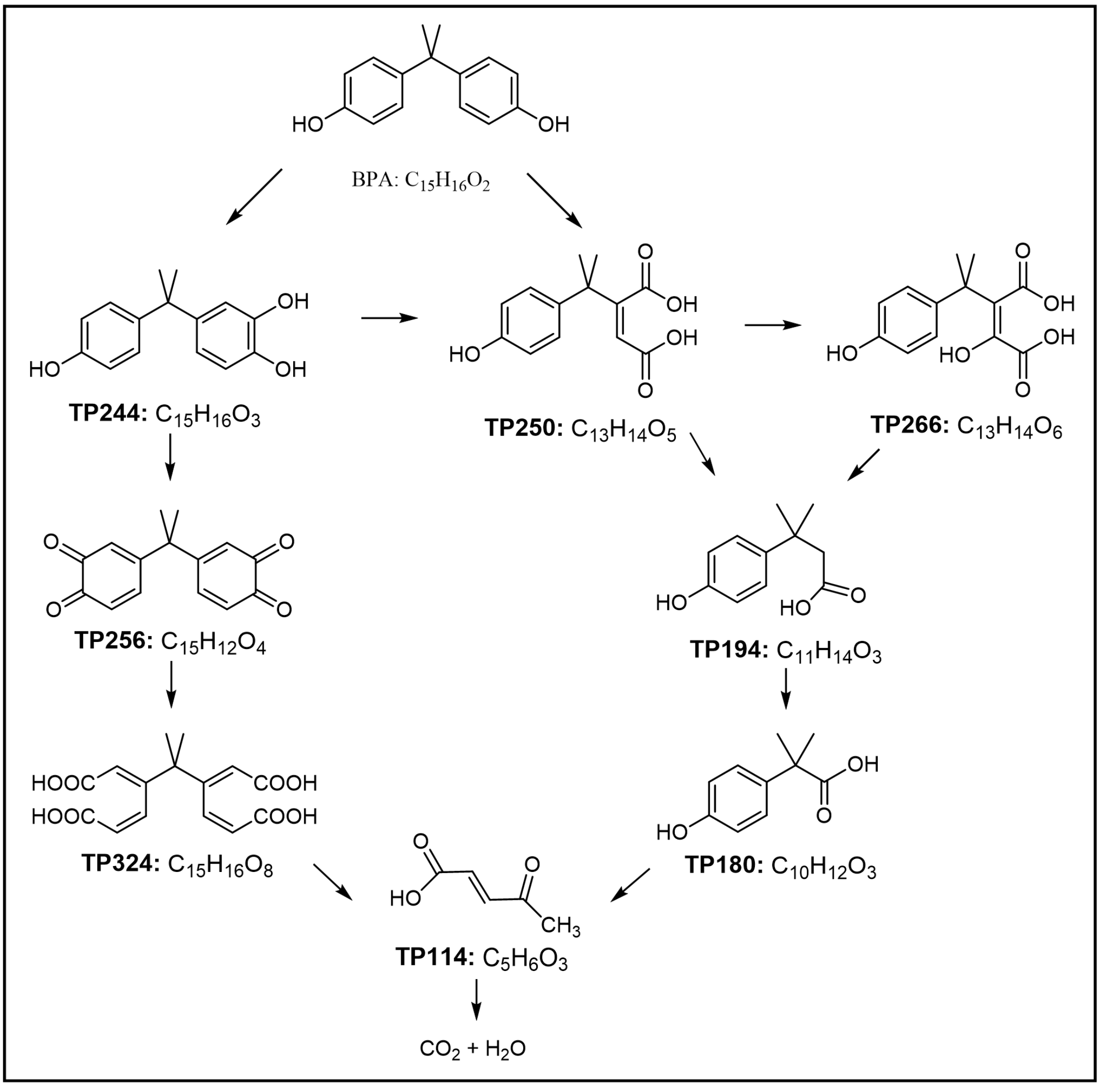

Supplementary Figure 37. Proposed transformation pathway of BPA in the Co_3O_4-*in*-CNT/PAA process. **TP244**, **TP250**, **TP266**, **TP256**, **TP194**, **TP324**, **TP180** and **TP114** represent transformation products, which are named for their relative molecular mass.

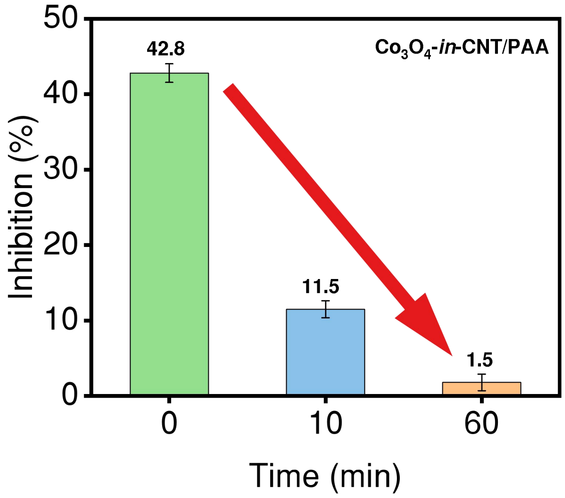

Supplementary Figure 38. Evolution of luminescence inhibition along the reaction. $[PAA]_0$ = 200 μM, $[Co_3O_4\text{-in-CNT}]_0$ = 0.1 g L^{-1}, $[BPA]_0$ = 100 μM, pH = 7.0. The error bars in the figures represent the standard deviations from triplicate tests.

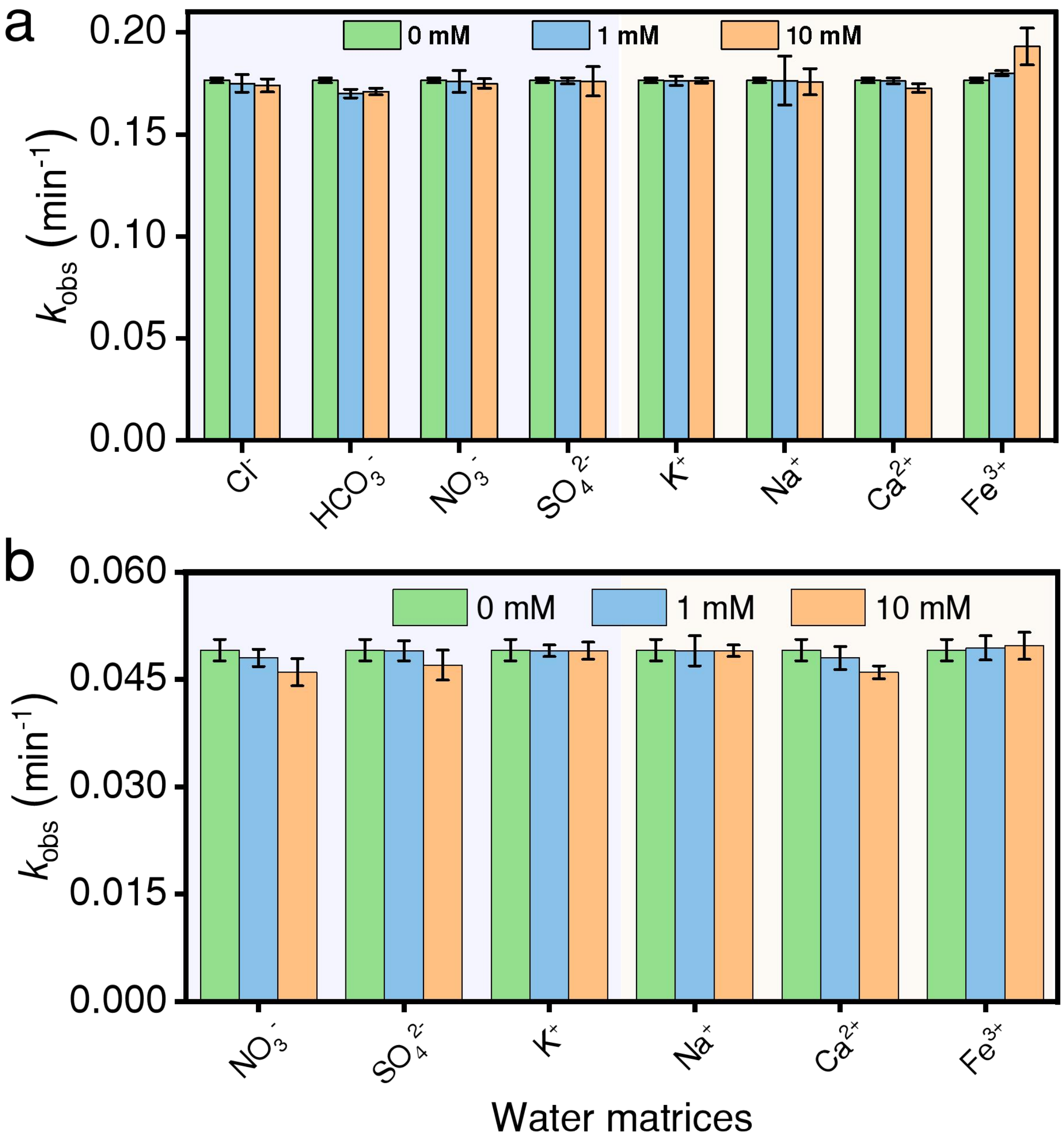

Supplementary Figure 39. The degradation rate of BPA in the (**a**) Co_3O_4-*in*-CNT/PAA (**b**) Co_3O_4-*out*-CNT/PAA system with the presence of different ions. $[PAA]_0$ = 200 μM, $[BPA]_0$ = 100 μM, $[Co_3O_4\text{-}in\text{-}CNT]_0$ = $[Co_3O_4\text{-}out\text{-}CNT]_0$ = 0.1 g L^{-1}, $[Cl^-]_0$ = $[HCO_3^-]_0$ = $[NO_3^-]_0$ = $[SO_4^{2-}]_0$ = $[K^+]_0$ = $[Na^+]_0$ = $[Ca^{2+}]_0$ = $[Fe^{3+}]_0$ = 0, 1, 10 mM, pH = 7.0. The error bars in the figures represent the standard deviations from triplicate tests.

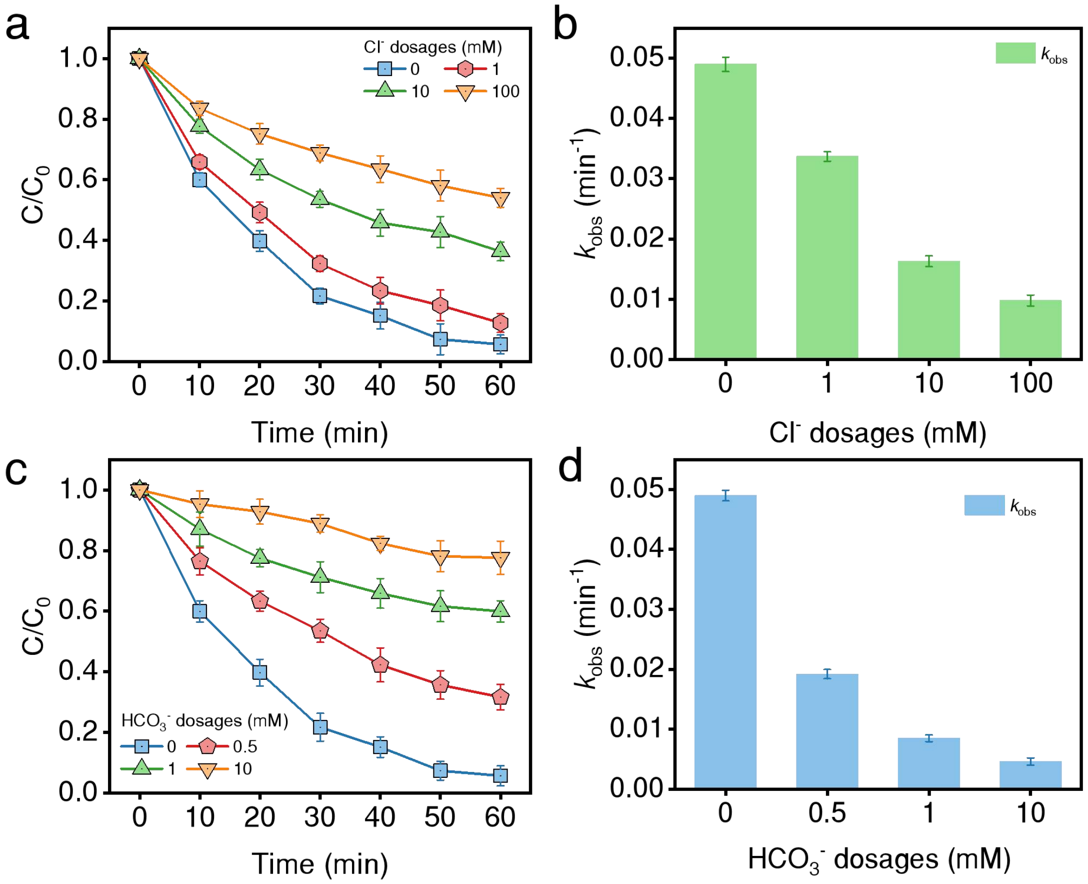

Supplementary Figure 40. Effects of (**a**) Cl^- and (**c**) HCO_3^- on BPA degradation in the Co_3O_4-*out*-CNT/PAA process; the k_{obs} of BPA with different (**b**) Cl^- and (d) HCO_3^- concentrations in the Co_3O_4-*out*-CNT/PAA process. $[PAA]_0$ = 200 μM, $[BPA]_0$ = 100 μM, $[Co_3O_4\text{-}out\text{-}CNT]_0$ = 0.1 g L^{-1}, $[Cl^-]_0$ = 1-100 mM, $[HCO_3^-]_0$ = 0.5-10 mM, pH = 7.0. The error bars in the figures represent the standard deviations from triplicate tests.

In the Co_3O_4-*in*-CNT/PAA system, the BPA was slightly suppressed in the real environmental water, such as lake water and secondary effluent from a municipal wastewater treatment plant. This might be due to the size exclusion effect of real water constituents, such as NOM and soluble microbial products, under spatial nanoconfinement. While in the Co_3O_4-*out*-CNT/PAA system, NOM, (bi)-carbonate, halide ions, and other coexisting ions with reducibility rapidly quench R-O•, thereby significantly inhibiting the removal of BPA.

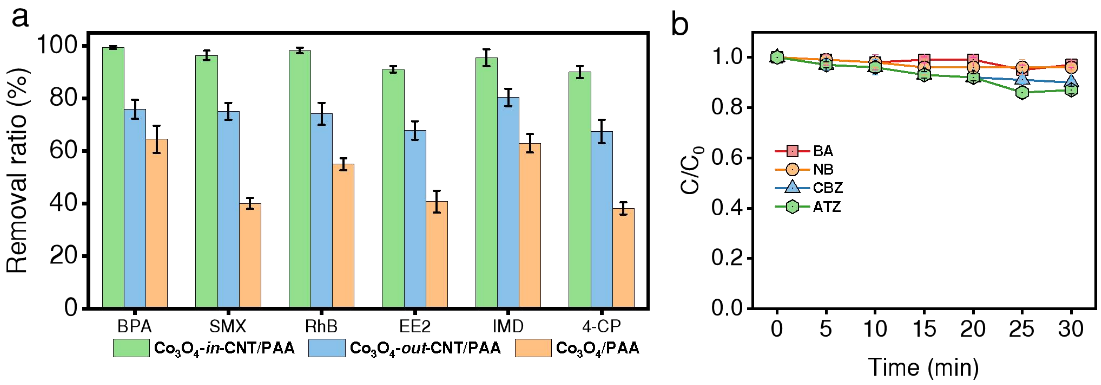

Supplementary Figure 41. (**a**) Degradation of various organic contaminants in different Fenton-like systems (BPA bisphenol A, SMX sulfamethoxazole, RhB Rhodamine B, EE2, 17α-ethinylestradiol, IMD imidacloprid, 4-CP 4-chlorophenol). (**b**) Degradation of contaminants with electron-withdrawing groups (i.e., NB nitrobenzene, BA benzoic acid, CBZ carbamazepine, ATZ atrazine) in the Co_3O_4-*in*-CNT/PAA system. $[PAA]_0$ = 200 μM, $[contaminants]_0$ = 20 μM, $[Co_3O_4\text{-}in\text{-}CNT]_0$ = 0.1 g L^{-1}, pH = 7.0. The error bars in the figures represent the standard deviations from triplicate tests.

Generally, pollutants were vulnerable to be degraded non-selectively by free radicals, whereas pollutants containing electron-donating groups (e.g. phenolic, dyes, some antibiotics, etc.) could be quickly oxidized by nonradical process.

Supplementary reference

1. Liu T, Chen J, Li N, Xiao S, Huang C-H, Zhang L, et al. Unexpected Role of Nitrite in Promoting Transformation of Sulfonamide Antibiotics by Peracetic Acid: Reactive Nitrogen Species Contribution and Harmful Disinfection Byproduct Formation Potential. *Environ. Sci. Technol.* **56**, 1300-1309 (2022).

2. Cai M, Sun P, Zhang L, Huang C-H. UV/Peracetic acid for degradation of pharmaceuticals and reactive species evaluation. *Environ. Sci. Technol.* **51**, 14217-14224 (2017).

3. Lee J, Gunten UV, Kim JH. Persulfate-based Advanced Oxidation: Critical Assessment of Opportunities and Roadblocks. *Environ. Sci. Technol.* **54**, 3064-3081 (2020).

4. Liu T, Zhang D, Yin K, Yang C, Luo S, Crittenden JC. Degradation of thiacloprid via unactivated peroxymonosulfate: The overlooked singlet oxygen oxidation. *Chem. Eng. J.* **388**, 124264 (2020).

5. Kim J, Huang C-H. Reactivity of peracetic acid with organic compounds: A critical review. *ACS ES&T Water.* **1**, 15-33 (2020).

6. Kim J, Zhang T, Liu W, Du P, Dobson JT, Huang C-H. Advanced Oxidation Process with Peracetic Acid and Fe (II) for Contaminant Degradation. *Environ. Sci. Technol.* **53**, 13312-13322 (2019).

7. Zhang L, Chen J, Zhang Y, Yu Z, Ji R, Zhou X. Activation of peracetic acid with cobalt anchored on 2D sandwich-like MXenes (Co@MXenes) for organic contaminant degradation: High efficiency and contribution of acetylperoxyl radicals. *Appl. Catal. B* **297**, 120475 (2021).

8. Wang J, Xiong B, Miao L, Wang S, Xie P, Wang Z, et al. Applying a novel advanced oxidation process of activated peracetic acid by $CoFe_2O_4$ to efficiently degrade sulfamethoxazole. *Appl. Catal. B* **280**, 119422 (2021).

9. Wu W, Tian D, Liu T, Chen J, Huang T, Zhou X, et al. Degradation of organic compounds by peracetic acid activated with Co_3O_4: A novel advanced oxidation process and organic radical contribution. *Chem. Eng. J.* **394**, 124938 (2020).

10. Liu B, Guo W, Jia W, Wang H, Si Q, Zhao Q, et al. Novel Nonradical Oxidation of Sulfonamide Antibiotics with Co (II)-Doped g-C_3N_4-Activated Peracetic Acid: Role of High-Valent Cobalt–Oxo Species. *Environ. Sci. Technol.* **55**, 12640-12651 (2021).

11. Wang J, Wan Y, Ding J, Wang Z, Ma J, Xie P, et al. Thermal Activation of Peracetic Acid in Aquatic Solution: The Mechanism and Application to Degrade Sulfamethoxazole. *Environ. Sci. Technol.* **54**, 14635-14645 (2020).

12. Kim J, Du P, Liu W, Luo C, Zhao H, Huang C-H. Cobalt/Peracetic Acid: Advanced Oxidation of Aromatic Organic Compounds by Acetylperoxyl Radicals. *Environ. Sci. Technol.* **54**, 5268-5278 (2020).

13. Chen H, Lin T, Zhang S, Xu H, Chen W. Novel Fe^{II}/EDDS/UV/PAA advanced oxidation process: Mechanisms and applications for naproxen degradation at neutral pH and low Fe^{II} dosage. *Chem. Eng. J.* **417**, 127896 (2020).

14. Wang Z, Wang J, Xiong B, Bai F, Wang S, Wan Y, et al. Application of cobalt/peracetic acid to degrade sulfamethoxazole at neutral condition: Efficiency and mechanisms. *Environ. Sci. Technol.* **54**, 464-475 (2019).

15. Chen S, Cai M, Liu Y, Zhang L, Feng L. Effects of water matrices on the degradation of naproxen by reactive radicals in the UV/peracetic acid process. *Water Res.* 2019, **150:** 153-161.

16. Zhang L, Chen J, Zhang Y, Xu Y, Zheng T, Zhou X. Highly efficient activation of peracetic acid by nano-CuO for carbamazepine degradation in wastewater: The significant role of H_2O_2 and evidence of acetylperoxy radical contribution. *Water Res.* **150**, 153-161 (2019).

17. Zhang Y, Zhang H, Ji H, Ma W, Chen C, Zhao J. Pivotal role and regulation of proton transfer in water oxidation on hematite photoanodes. *J. Am. Chem. Soc.* **138**, 2705-2711 (2016).

18. Zhang Y-J, Huang G-X, Winter LR, Chen J-J, Tian L, Mei S-C, et al. Simultaneous nanocatalytic surface activation of pollutants and oxidants for highly efficient water decontamination. *Nat. Commun.* **13**, 1-13 (2022).

19. McDowell MS, Bakac A, Espenson JH. A convenient route to superoxide ion in aqueous solution. *Inorg. Chem.* 1983, **22**, 847-848 (1983).

20. Ma J, Zhou H, Yan S, Song W. Kinetics studies and mechanistic considerations on the reactions of superoxide radical ions with dissolved organic matter. *Water Res.* **149**, 56-64 (2019).

21. Garg S, Rose AL, Waite TD. Photochemical production of superoxide and hydrogen peroxide from natural organic matter. *Geochim. Cosmochim. Acta* **75**, 4310-4320 (2011).

22. Fujii M, Rose AL, Waite TD, Omura T. Superoxide-mediated dissolution of amorphous ferric oxyhydroxide in seawater. *Environ. Sci. Technol.* **40**, 880-887 (2006).

23. Kozmér Z, Arany E, Alapi T, Takács E, Wojnárovits L, Dombi A. Determination of the

rate constant of hydroperoxyl radical reaction with phenol. *Radiation Physics and Chemistry*, **102**, 135-138 (2014).

24. Yang Z, Qian J, Yu A, Pan B. Singlet oxygen mediated iron-based Fenton-like catalysis under nanoconfinement. *Proc. Natl. Acad. Sci.* **116**, 6659-6664 (2019).

25. Zhou Y, Jiang J, Gao Y, Ma J, Pang S-Y, Li J, et al. Activation of peroxymonosulfate by benzoquinone: a novel nonradical oxidation process. *Environ. Sci. Technol.* **49**, 12941-12950 (2015).

26. Halladja S, Ter Halle A, Aguer J-P, Boulkamh A, Richard C. Inhibition of humic substances mediated photooxygenation of furfuryl alcohol by 2, 4, 6-trimethylphenol. Evidence for reactivity of the phenol with humic triplet excited states. *Environ. Sci. Technol.* **41**, 6066-6073 (2017).

27. Buxton GV, Greenstock CL, Helman WP, Ross AB. Critical Review of rate constants for reactions of hydrated electrons, hydrogen atoms and hydroxyl radicals ($\cdot OH/\cdot O^-$) in Aqueous Solution. *J. Phys. Chem. Ref. Data* **17**, 513-886 (1988).

28. Yang Y, Banerjee G, Brudvig GW, Kim JH, Pignatello JJ. Oxidation of Organic Compounds in Water by Unactivated Peroxymonosulfate. *Environ. Sci. Technol.* **52**, 5911-5919 (2018).

29. Liu T, Yin K, Liu C, Luo J, Crittenden J, Zhang W, et al. The role of reactive oxygen species and carbonate radical in oxcarbazepine degradation via UV, UV/H_2O_2: kinetics, mechanisms and toxicity evaluation. *Water Res.* **147**, 204-213 (2018).

30. Li H, Shan C, Pan B. Fe (III)-doped g-C_3N_4 mediated peroxymonosulfate activation for selective degradation of phenolic compounds via high-valent iron-oxo species. *Environ. Sci. Technol.* 2018, **52**, 2197-2205 (2018).

31. Zhou X, Ke M-K, Huang G-X, Chen C, Chen W, Liang K, et al. Identification of Fenton-like active Cu sites by heteroatom modulation of electronic density. *Proc. Natl. Acad. Sci.* **119**, e2119492119 (2022).

32. Wu L, Li B, Li Y, Fan X, Zhang F, Zhang G, et al. Preferential Growth of the Cobalt (200) Facet in Co@N–C for Enhanced Performance in a Fenton-like Reaction. *ACS Catal.* **11**, 5532-5543 (2021).

33. Liu H, Han J, Yuan J, Liu C, Wang D, Liu T, et al. Deep dehalogenation of florfenicol using crystalline CoP nanosheet arrays on a Ti plate via direct cathodic reduction and atomic H. *Environ. Sci. Technol.* **53**, 11932-11940 (2019).

34. Yang Z, Qian J, Shan C, Li H, Yin Y, Pan B. Toward selective oxidation of contaminants

in aqueous systems. *Environ. Sci. Technol.* **55**, 14494-14514 (2021).

35. Zhou S, Yu Y, Zhang W, Meng X, Luo J, Deng L, et al. Oxidation of Microcystin-LR via Activation of Peroxymonosulfate Using Ascorbic Acid: Kinetic Modeling and Toxicity Assessment. *Environ. Sci. Technol.* **52**, 4305-4312 (2018).

36. Yin K, Li T, Zhang T, Zhang Y, Yang C, Luo S. Degradation of organic filter 2-Phenylbenzidazole-5-Sulfonic acid by light-driven free chlorine process: Reactive species and mechanisms. *Chem. Eng. J.* **430**, 132684 (2021).

37. Zhang S, Sun M, Hedtke T, Deshmukh A, Zhou X, Weon S, et al. Mechanism of Heterogeneous Fenton Reaction Kinetics Enhancement under Nanoscale Spatial Confinement. *Environ. Sci. Technol.* **54**, 10868-10875 (2020).

38. Chu C, Yang J, Huang D, Li J, Wang A, Alvarez PJ, et al. Cooperative pollutant adsorption and persulfate-driven oxidation on hierarchically ordered porous carbon. *Environ. Sci. Technol.* **53**, 10352-10360 (2019).

39. Zhang S, Hedtke T, Zhu Q, Sun M, Weon S, Zhao Y, et al. Membrane-Confined Iron Oxychloride Nanocatalysts for Highly Efficient Heterogeneous Fenton Water Treatment. *Environ. Sci. Technol.* **54**, 10868-10875 (2020).

40. Luo J, Liu T, Zhang D, Yin K, Wang D, Zhang W, et al. The individual and Co-exposure degradation of benzophenone derivatives by UV/H_2O_2 and UV/PDS in different water matrices. *Water Res.* **159**, 102-110 (2019).

41. Wang Z, Wang J, Xiong B, Bai F, Wang S, Wan Y, et al. Application of cobalt/peracetic acid to degrade sulfamethoxazole at neutral condition: Efficiency and mechanisms. *Environ. Sci. Technol.* **54**, 464-475 (2019).

42. Oh W-D, Lua S-K, Dong Z, Lim T-T. High surface area DPA-hematite for efficient detoxification of bisphenol A via peroxymonosulfate activation. *J. Mater. Chem. A.* **2**, 15836-15845 (2014).

43. Oh W-D, Dong Z, Hu Z-T, Lim T-T. A novel quasi-cubic $CuFe_2O_4$–Fe_2O_3 catalyst prepared at low temperature for enhanced oxidation of bisphenol A via peroxymonosulfate activation. *J. Mater. Chem. A.* **3**, 22208-22217 (2015).

44. Huang G-X, Wang C-Y, Yang C-W, Guo P-C, Yu H-Q. Degradation of Bisphenol A by Peroxymonosulfate Catalytically Activated with $Mn_{1.8}Fe_{1.2}O_4$ Nanospheres: Synergism between Mn and Fe. *Environ. Sci. Technol.* 2017, **51**, 12611-12618 (2017).

45. Li X, Wang Z, Zhang B, Rykov AI, Ahmed MA, Wang J. $FexCo_3{-}xO_4$ nanocages derived from nanoscale metal–organic frameworks for removal of bisphenol A by activation of

peroxymonosulfate. *Appl. Catal. B* **181**, 788-799 (2016).

46. Li X, Huang X, Xi S, Miao S, Ding J, Cai W, et al. Single cobalt atoms anchored on porous N-doped graphene with dual reaction sites for efficient Fenton-like catalysis. *J. Am. Chem. Soc.* **140**, 12469-12475 (2018).

47. Zhu Y, Zhu R, Xi Y, Xu T, Yan L, Zhu J, et al. Heterogeneous photo-Fenton degradation of bisphenol A over Ag/AgCl/ferrihydrite catalysts under visible light. *Chem. Eng. J.* 2018, **346**, 567-577 (2018).

48. Lyu L, Yan D, Yu G, Cao W, Hu C. Efficient Destruction of Pollutants in Water by a Dual-Reaction-Center Fenton-like Process over Carbon Nitride Compounds-Complexed Cu(II)-$CuAlO_2$. *Environ. Sci. Technol.* **52**, 4294-4304 (2018).

49. Gao Y, Wu T, Yang C, Ma C, Zhao Z, Wu Z, et al. Activity Trends and Mechanisms in Peroxymonosulfate-Assisted Catalytic Production of Singlet Oxygen over Atomic Metal-N-C Catalysts. *Angew. Chem. Int. Ed.* 2021, **60**, 22513-22521 (2021).

50. Wang W, Liu Y, Yue Y, Wang H, Cheng G, Gao C, et al. The Confined Interlayer Growth of Ultrathin Two-Dimensional Fe_3O_4 Nanosheets with Enriched Oxygen Vacancies for Peroxymonosulfate Activation. *ACS Catal.* **11**, 11256-11265 (2021).

51. Li X, Ao Z, Liu J, Sun H, Rykov AI, Wang J. Topotactic Transformation of Metal–Organic Frameworks to Graphene-Encapsulated Transition-Metal Nitrides as Efficient Fenton-like Catalysts. *ACS Nano* **10**, 11532-11540 (2016).

52. Zhang M, Xiao C, Yan X, Chen S, Wang C, Luo R, et al. Efficient removal of organic pollutants by metal–organic framework derived Co/C yolk–shell nanoreactors: Size-exclusion and confinement effect. *Environ. Sci. Technol.* **54**, 10289-10300 (2020).

53. Kim J, Zhang T, Liu W, Du P, Dobson JT, Huang CH. Advanced Oxidation Process with Peracetic Acid and Fe(II) for Contaminant Degradation. *Environ. Sci. Technol.* **53**, 13312-13322 (2019).

54. Rokhina EV, Makarova K, Golovina EA, Van As H, Virkutyte J. Free radical reaction pathway, thermochemistry of peracetic acid homolysis, and its application for phenol degradation: spectroscopic study and quantum chemistry calculations. *Environ. Sci. Technol.* **44**, 6815-6821 (2010).

55. Rokhina EV, Makarova K, Lahtinen M, Golovina EA, Van As H, Virkutyte J. Ultrasound-assisted MnO_2 catalyzed homolysis of peracetic acid for phenol degradation: The assessment of process chemistry and kinetics. *Chem. Eng. J.* **221**, 476-486 (2013).

56. Chalier F, Tordo P. 5-Diisopropoxyphosphoryl-5-methyl-1-pyrroline N-oxide, DIPPMPO,

a crystalline analog of the nitrone DEPMPO: synthesis and spin trapping propertiesElectronic supplementary information (ESI) available: Tables of crystallographic data. *J. Chem. Soc.* **2**, 2110-2117 (2002).

57. Frederick A. Villamena, Christopher M. Hadad, Zweier JL. Kinetic Study and Theoretical Analysis of Hydroxyl Radical Trapping and Spin Adduct Decay of Alkoxycarbonyl and Dialkoxyphosphoryl Nitrones in Aqueous Media. *J. Phys. Chem. A.* **107**, 4407-4414 (2003).

58. Fu F, Wang C, Wang Q, Martinez-Villacorta AM, Escobar A, Chong H, et al. Highly selective and sharp volcano-type synergistic Ni_2Pt@ ZIF-8-catalyzed hydrogen evolution from ammonia borane hydrolysis. *J. Am. Chem. Soc.* **140**, 10034-10042 (2018).

59. Wang Y, Zhou T, Jiang K, Da P, Peng Z, Tang J*, et al.* Reduced mesoporous Co_3O_4 nanowires as efficient water oxidation electrocatalysts and supercapacitor electrodes. *Adv. Energy. Mater.* 2014, **4**, 1400696 (2014).

60. Liu B, Song W, Wu H, Liu Z, Teng Y, Sun Y, et al. Degradation of norfloxacin with peroxymonosulfate activated by nanoconfinement Co_3O_4@CNT nanocomposite. *Chem. Eng. J.* **398**, 125498 (2020).

61. Zhuang L, Ge L, Yang Y, Li M, Jia Y, Yao X, et al. Ultrathin iron-cobalt oxide nanosheets with abundant oxygen vacancies for the oxygen evolution reaction. *Adv. Mater.* **29**, 1606793 (2017).

62. Wu Z, Xiong Z, Liu R, He C, Liu Y, Pan Z, et al. Pivotal roles of N-doped carbon shell and hollow structure in nanoreactor with spatial confined Co species in peroxymonosulfate activation: Obstructing metal leaching and enhancing catalytic stability. *J. Hazard. Mater.* **427**, 128204 (2022).

63. Chen Z, Guan Z, Li M, Yang Q, Li C. Enhancement of the performance of a platinum nanocatalyst confined within carbon nanotubes for asymmetric hydrogenation. *Angew. Chem. Int. Ed.* **123**, 5015-5019 (2011).

64. Liu B, Guo W, Jia W, Wang H, Zheng S, Si Q, et al. Insights into the oxidation of organic contaminants by Co (II) activated peracetic acid: The overlooked role of high-valent cobalt-oxo species. *Water Res.* **201**, 117313 (2021).

65. Ji J, Yan Q, Yin P, Mine S, Matsuoka M, Xing M. Defects on CoS_2−x: Tuning Redox Reactions for Sustainable Degradation of Organic Pollutants. *Angew. Chem. Int. Ed.* **133**, 2939-2944 (2021).

66. Chen F, Liu LL, Wu JH, Rui XH, Chen JJ, Yu Y. Single-Atom Iron Anchored Tubular g-C_3N_4 Catalysts for Ultrafast Fenton-Like Reaction: Roles of High-Valent Iron-Oxo

Species and Organic Radicals. *Adv. Mater.* **34**, 2202891 (2022).
